# Generalizing the first-difference correlated random walk for marine animal movement data

**DOI:** 10.1038/s41598-019-40405-z

**Published:** 2019-03-08

**Authors:** Christoffer Moesgaard Albertsen

**Affiliations:** National Institute of Aquatic Resources, Technical University of Denmark, Kemitorvet 201, DK-2800 Kgs., Lyngby, Denmark

## Abstract

Animal telemetry data are often analysed with discrete time movement models. These models are defined with regular time steps. However, telemetry data from marine animals are observed irregularly. To account for irregular data, a time-irregularised first-difference correlated random walk model with drift is introduced. The model generalizes the commonly used first-difference correlated random walk with regular time steps by allowing irregular time steps, including a drift term, and by allowing different autocorrelation in the two coordinates. The model is applied to data from a ringed seal collected through the Argos satellite system, and is compared to related movement models through simulations. Accounting for irregular data in the movement model results in accurate parameter estimates and reconstruction of movement paths. Further, the introduced model can provide more accurate movement paths than the regular time counterpart. Extracting accurate movement paths from uncertain telemetry data is important for evaluating space use patterns for marine animals, which in turn is crucial for management. Further, handling irregular data directly in the movement model allows efficient simultaneous analyses of several animals.

## Introduction

Understanding animal movement behaviour, space use patterns, and response to the environment relies on modelling animal telemetry data. Collecting telemetry data in marine environments is difficult: satellite systems require animals to surface, storage tags require recapture, and acoustic networks require a dense array of receivers. Nonetheless, the scope and scales of movement studies are continuously increasing with rapid technological advances in tracking devices^1^.

In spite of technological advances leading to more accurate measurements and larger datasets, data from satellite tags have inherent measurement errors. Systems such as Fastloc GPS and Argos can only record data when the animal is above water, with an accuracy that depends on the surface time and diving behaviour^2^. Typically, this results in data observed at irregular time steps with considerable uncertainty, which makes state-space models a valuable framework for analysing the data.

State-space models naturally separate the movement process from the measurement mechanism. True animal locations are explicitly modelled separately from the observed locations. True locations are modelled directly by a stochastic process, while an observed location is modelled by the conditional distribution given the true location. Hence, the movement models developed can be combined with telemetry data from any source, for example, GPS, Argos, light-based, or acoustic tags. However, inference in non-linear or non-Gaussian state-space models can be challenging. Since the true locations are not observed, maximum likelihood estimation in state-space models requires obtaining the marginal distribution of the observations. The marginal distribution is obtained by calculating the joint distribution of true and observed locations and integrating over all possible true locations that could have been observed. Recent software such as AD Model Builder^3^ and Template Model Builder (TMB^4^) utilizes the Laplace approximation to rapidly approximate the marginal distribution of observations in state-space models. While care is needed to ensure the approximation is appropriate, this framework is generally applicable. For instance, TMB has been used for both discrete time^5,6^ and continuous time^7–9^ movement models. Alternatively, the state-space can be discretized and the resulting hidden Markov model can be used for inference^10^.

Although continuous time movement models can handle irregular data, discrete time models are often thought of as more intuitive^11^. Further, continuous time models can be difficult to construct or extend analytically, because they are often formulated through stochastic differential equations. Whereas continuous time models describe the instantaneous change, discrete time models describe the change between two observations. The change between observations is often modelled by the distance and the turning angle (e.g.^12–17^), which is considered a natural way to represent movement^18^. In discrete time models with regular time steps, irregular data can be handled by modifying the observational model to interpolate between states^13^.

To facilitate the analysis of populations or meta-analysis of individuals, the model parameters for different individuals must be on the same scale. If the interpretation of parameter values depends on the chosen time steps between estimated locations^11^, the values must be corrected before comparison is possible. Likewise, estimating population parameters based on individuals either requires movement models that use the same time steps for all individuals (e.g.^19^), which can be sub-optimal, or movement models that handle irregular data directly.

The first-difference correlated random walk (DCRW^13^) models animal movement as a discrete time first order autoregressive process on the difference between consecutive locations. The model includes a rotation parameter which allows tortuous and circular movement patterns. This is similar to the mean turning angle included in many discrete time models (e.g.^12,15–17^). A continuous time version of the DCRW without rotation is the continuous time correlated random walk (CTCRW^20^). The CTCRW models the velocity of an animal by an Ornstein-Uhlenbeck process: the continuous time counterpart of the first order autoregressive process. Recently, an irregular time version of the DCRW was introduced without rotation including a time-varying parameter instead^6^.

In this paper, a generalization of the DCRW is introduced. The generalization of the DCRW is trifold: the model allows irregular time steps, a drift vector is included, and different autocorrelations in the two coordinates can be used. As a by-product, it is shown how the parameters in the DCRW model depend on the selected time steps. Further, a formula for transforming them to a time-invariant scale is obtained. The generalized DCRW is introduced as a discretization of a stochastic differential equation for the animal’s velocity and location. Further, the close relationship between the generalized DCRW and a generalization of the CTCRW is shown. Through simulation studies, the generalized DCRW is shown to perform well compared to the CTCRW and DCRW. Finally, the applicability and extendability of the model are illustrated through a real animal movement dataset.

## Materials and Methods

### Movement model

The generalization of the DCRW movement model is constructed through a stochastic differential equation (SDE) for the velocity. From this SDE, the location process is the integrated velocity process. To obtain the time-irregular movement model, the SDE is solved analytically while the location process is a discrete time approximation.

#### SDE for velocity

The bivariate SDE for the velocity, *V*_*t*_, of an animal is

1 $$\begin{array}{rcl}{\rm{d}}{V}_{t} & = & -(\begin{array}{cc}-\mathrm{log}\,{\gamma }_{1} & \theta \\ -\theta  & -\mathrm{log}\,{\gamma }_{2}\end{array})({V}_{t}-\mu ){\rm{d}}t+Sd{B}_{t}\\  & = & -{\rm{\Theta }}({V}_{t}-\mu ){\rm{d}}t+Sd{B}_{y}\end{array}$$

with initial condition ${V}_{0}={v}_{0}\in {{\mathbb{R}}}^{2}$. In this model, *γ*_1_ and *γ*_2_ are autocorrelation parameters, *μ* is a vector of mean velocity parameters, and *θ* is a rotation parameter related to the mean turning angle of the DCRW model. The matrix *S* is a lower triangular matrix determining the covariance of the changes in velocity. Diagonal elements of *S* must be positive. When off-diagonal elements are zero, changes in velocity are independent between coordinates and the diagonal is their standard deviation. An overview of the parameters is given in Table 1. Animals moving persistently in one direction will have a high autocorrelation in the velocity and a rotation parameter close to zero, while a lower autocorrelation in the velocity and a rotation parameter different from zero results in tortuous movement trajectories with circular patterns and frequent changes in velocity (e.g.^13,21^). When *γ*_1_ = *γ*_2_ and *S* = *σI*_2_, where *I*_*n*_ denotes an *n* × *n* identity matrix, the model is identical to the rotational-advective correlated velocity model discussed by Gurarie *et al*.^22^. In this case, *θ* can be interpreted as a mean radial velocity^22^.

**Table 1:** Overview of movement model parameters.

Parameter	Values	Interpretation
*V*_*t*_	${{\mathbb{R}}}^{2}$	Bivariate velocity process at time *t*
*v*_0_	${{\mathbb{R}}}^{2}$	Initial velocity
*X*_*t*_	${{\mathbb{R}}}^{2}$	Bivariate location process at time *t*
*B*_*t*_	${{\mathbb{R}}}^{2}$	Bivariate standard Brownian motion at time *t*
*γ*_1_	(0; 1]	Autocorrelation in the first coordinate
*γ*_2_	(0; 1]	Autocorrelation in the second coordinate
*θ*	${\mathbb{R}}$	A combination of turning angle and number of turns per time unit
*μ*	${{\mathbb{R}}}^{2}$	Vector of mean velocities resulting in drifted movement
*S*_11_	(0, ∞)	Parameter determining the velocity process variance in the first coordinate
*S*_22_	(0, ∞)	Parameter determining the velocity process variance in the first coordinate
*S*_21_	(−∞, ∞)	Parameter determining the velocity process correlation
*S*_12_	0	The *S* matrix must be lower triangular to be identifiable
Σ	*SS* ^*T*^	
Θ	$$(\begin{array}{cc}-log{\gamma }_{1} & \theta \\ -\theta & -log{\gamma }_{2}\end{array})$$	
Δ_*i*_	(0, ∞)	Time between location estimate *i* − 1 and *i*

The analytical solution to the SDE is a stochastic process with Gaussian increments (see e.g. Supplemental Material S1). The mean of an increment is

$$E({V}_{t}|{V}_{s})={e}^{-{\rm{\Theta }}(t-s)}{V}_{s}+(I-{e}^{-{\rm{\Theta }}(t-s)})\mu ,\,s < t$$

where $\mu ={({\mu }_{1},{\mu }_{2})}^{T}$, while the covariance is

$${\rm{vec}}(Var({V}_{t}|{V}_{s}))={\rm{vec}}(C)-{e}^{-{\rm{\Theta }}(t-s)}\otimes {e}^{-{\rm{\Theta }}(t-s)}{\rm{vec}}(C),\,s < t$$

where ${\rm{vec}}(C)={({\rm{\Theta }}\oplus {\rm{\Theta }})}^{-1}\,{\rm{vec}}({\rm{\Sigma }})$, ${\rm{\Sigma }}=S{S}^{T}$, ⊕ denotes the Kronecker sum, $A\oplus B=A\otimes {I}_{B}+{I}_{A}\otimes B$, and vec is an operator that stacks the columns of a matrix to a vector. Based on the velocity, the location process, *X*_*t*_, follows the SDE

2 $${\rm{d}}{X}_{t}={V}_{t}{\rm{d}}t$$

#### Discretizing the SDE for locations

Considering the *N* predetermined time points ${\{{t}_{i}\}}_{i\in \{\mathrm{1,}\ldots ,N\}}$, an Euler–Maruyama approximation to the location process is obtained by

$${X}_{{t}_{i}}={X}_{{t}_{i-1}}+{V}_{{t}_{i-1}}{{\rm{\Delta }}}_{i}$$

where ${{\rm{\Delta }}}_{i}={t}_{i}-{t}_{i-1}$ is the length of the time step. Inserting the expression for ${V}_{{t}_{i-1}}$, the Generalized first-Difference Correlated Random Walk (GDCRW) is obtained:

3 $${X}_{{t}_{i}}={X}_{{t}_{i-1}}+{{\rm{\Delta }}}_{i}{e}^{-{{\rm{\Theta }}{\rm{\Delta }}}_{i-1}}({X}_{{t}_{i-1}}-{X}_{{t}_{i-2}})/{{\rm{\Delta }}}_{i-1}+{{\rm{\Delta }}}_{i}({I}_{2}-{e}^{-{{\rm{\Theta }}{\rm{\Delta }}}_{i-1}})\mu +{{\rm{\Delta }}}_{i}{\varepsilon }_{{t}_{i}}$$

The error terms, ${\varepsilon }_{{t}_{i}}$, follow a zero mean normal distribution with variance

$$Var({\varepsilon }_{{t}_{i}})=C-{e}^{-{{\rm{\Theta }}{\rm{\Delta }}}_{i}}C{e}^{-{{\rm{\Theta }}}^{T}{{\rm{\Delta }}}_{i}}$$

with vec(*C*) as defined in Subsection **(SDE for velocity)**.

When the animal movement is continuous in time, the GDCRW model provides a discrete time approximation. The quality of the approximation depends on the time between locations of the movement process; that is, the predetermined time points ${\{{t}_{i}\}}_{i\in \mathrm{\{1,}\ldots ,N\}}$. The time points do not need to correspond directly to the time of observations. Therefore, the quality of the approximation can be improved by adding additional locations between observations, thereby making the time discretization finer. The additional locations must be integrated out of the likelihood. When the process is observed directly, this can be done by simulation^23,24^. In a state-space model, this simply corresponds to adding estimated locations without observations, since the entire location process is unobserved. Consequently, the additional estimated locations can be integrated out of the likelihood in the same way as the remaining process, for instance, with the Laplace approximation, filters or MCMC. In both cases, the approximation of the transition density between observations is improved, while only the marginal likelihood of observations is used for inference.

#### Special cases

The GDCRW model is closely related to four other models: The CTCRW, the DCRW, the modified DCRW by Auger-Méthé *et al*.^6^, and a random walk.

#### Relation to the CTCRW

When the turning-angle parameter *θ* = 0 and the increment covariance *S* is diagonal, the velocity model, *V*_*t*_, is identical to the velocity model of the CTCRW; however, the location models differ. While the location model of the CTCRW is an analytical solution to the two univariate integrated velocity models, the GDCRW is a discretization of the more general bivariate velocity model. Hence, when *θ* = 0 and *S* is a diagonal matrix, the discrete time GDCRW converges to the continuous time CTCRW as the time steps tend to zero. When *θ* ≠ 0 or *S* is not diagonal, the GDCRW converges to a generalization of the CTCRW when the length of the time steps approaches zero.

#### Relation to the DCRW

For regularly observed data, ${{\rm{\Delta }}}_{i}={\rm{\Delta }}$, with *μ* = 0 and *γ* = *γ*_1_ = *γ*_2_,

$${e}^{-{\rm{\Theta }}{\rm{\Delta }}}=(\begin{array}{cc}\cos (\theta {\rm{\Delta }}) & -\sin (\theta {\rm{\Delta }})\\ \sin (\theta {\rm{\Delta }}) & \cos (\theta {\rm{\Delta }})\end{array}){\gamma }^{{\rm{\Delta }}}$$

Hence, the movement model simplifies to

4 $${X}_{i}={X}_{i-1}+(\begin{array}{cc}\cos (\theta {\rm{\Delta }}) & -\sin (\theta {\rm{\Delta }})\\ \sin (\theta {\rm{\Delta }}) & \cos (\theta {\rm{\Delta }})\end{array}){\gamma }^{{\rm{\Delta }}}({X}_{i-1}-{X}_{i-2})+{\rm{\Delta }}{\varepsilon }_{i},$$

which is the movement model of Jonsen *et al*.^13^ when Δ = 1. Therefore, the GDCRW generalizes the DCRW in three ways: the restriction of regular time steps is relaxed, a drift vector is added, and different autocorrelations in the two coordinates are allowed. Further, equation (4) allows scaling the DCRW parameters to a common time scale. That is, if parameters $\tilde{\gamma }$ and $\tilde{\theta }$ are obtained from a DCRW model fitted with time steps $\tilde{{\rm{\Delta }}}$, scale-independent parameters are obtained by $\gamma ={\tilde{\gamma }}^{\mathrm{1/}\tilde{\Delta }}$ and $\theta =\tilde{\theta }/\tilde{\Delta }$. Being able to scale the DCRW parameters to a common scale allows comparison between model fits in meta or population studies even if different time scales are used.

#### Relation to the modified DCRW

Recently, Auger-Méthé *et al*.^6^ proposed a modified version of the DCRW for Fastloc-GPS data. The modified DCRW allowed irregularly observed data and included a time-varying autocorrelation parameter, *γ*_*t*_. Ignoring the time-varying autocorrelation, the modified DCRW can be obtained as a special case of the GDCRW introduced here by letting *γ*_1_ = *γ*_2_ = *γ*_*t*_, *μ* = 0, *θ* = 0, and by letting Σ be a diagonal matrix. Note, however, that the two models differ slightly in parameterization. In the modified DCRW, *γ*_*t*_ is not scaled by the time differences and the variance of ${\varepsilon }_{{t}_{i}}$ is parameterized as a diagonal matrix $diag({\sigma }_{1}^{2},{\sigma }_{2}^{2})$. The GDCRW can easily be extended to have a time-varying autocorrelation parameter in the same way as Auger-Méthé *et al*.^6^.

#### Relation to a random walk

Finally, the GDCRW can be reduced to a random walk model. From equation (3) it follows that when the autocorrelation parameters *γ*_1_ and *γ*_2_ tend to zero, the model is reduced to a random walk. This is also the case for the CTCRW, DCRW, and modified DCRW. If $\mu \ne 0$, it will be a random walk with drift.

### Measurement equation

Besides the movement process describing the true, latent locations, ${\{{X}_{{t}_{i}}\}}_{i\in \mathrm{\{1,}\ldots ,N\}}$, a measurement equation is needed to form a state-space model. For the DCRW, measurement errors are often included by linearly interpolating between the locations. For an observation ${Y}_{{s}_{j}}$ at time *s*_*j*_,

$${Y}_{{s}_{j}}=\mathrm{(1}-q){X}_{{t}_{i}}+q{X}_{{t}_{i+1}}+{\eta }_{j}$$

such that ${t}_{i}\le {s}_{j} < {t}_{i+1}$, $q=\frac{{s}_{j}-{t}_{i}}{{{\rm{\Delta }}}_{i+1}}$, and ${\eta }_{i}$ is a zero mean bivariate random variable. However, when the movement model allows irregular time steps, it is more natural to ensure that the estimated locations align with the observations, such that the measurement equation simplifies to

$${Y}_{{s}_{j}}={X}_{{s}_{j}}+{\eta }_{j}$$

Then the observation at time *s*_*j*_ does not depend on an estimated location in the future. Note that the state-space model can include additional estimated locations between observations. These estimated locations only contributes to the likelihood function through the movement process (see Supplemental Material S2). Below, ${\eta }_{j}$ will either be modelled as a bivariate normal distribution^20^ or a bivariate t-distribution^7^.

## Simulation Studies

To evaluate the GDCRW, three simulation studies were conducted. The first simulation study evaluated the GDCRW against the CTCRW when the latter was the true movement model. The second study compared the GDCRW to the DCRW when a continuous time version of the DCRW was the true model. Finally, the third study investigated the effect of measurement error on the choice of time step lengths in the GDCRW. The simulation studies only considered the main movement parameters. Variance parameters were not considered since they were related to the coordinate system used. For the CTCRW, the main movement parameter was the autocorrelation. For the DCRW, the main movement parameters were the mean turning angle and the autocorrelation.

The first simulation study was conducted to illustrate that the GDCRW could adequately reconstruct movement trajectories and parameter estimates from the CTCRW model. Likewise, the second simulation study was included to show that the GDCRW could recover parameter estimates from a continuous time version of the DCRW, including the rotation parameter not present in the CTCRW model. In contrast, the DCRW model itself had to be combined with equation (4) to recover the parameter estimates. Finally, the third simulation study was carried out to show that the irregular time steps of the GDCRW was better at reconstructing continuous time movement trajectories than the regular time steps of the DCRW, especially when measurement error was low.

In all three simulation studies, location trajectories were simulated from the continuous time model defined by equations (1) and (2). In the first simulation study, the GDCRW was therefore a discretized version of the true model, while the CTCRW was the true model. In the second and third simulation studies, the DCRW and GDCRW were different discretizations of the true model. The Euler-Maruyama method was used to simulate from the model. To emulate continuous time movement, the Euler-Maruyama method was used with 200 additional simulated locations between observations. Time steps between observations were simulated from a mixture of an exponential and a normal distribution (see Supplemental Material S3) to ensure both short and long time steps between observations. All models in the simulation studies were fitted to the data by maximum likelihood through the Laplace approximation using the R (version 2018-03-21 r74432^25^) package argosTrack (version 1.2.2^26^), which builds on the package TMB (version 1.7.14^4^) (R code is available as supplementary material).

### Evaluating the GDCRW against the CTCRW

Since the GDCRW location process is constructed as an Euler–Maruyama discrete time approximation to an underlying continuous time model, increasing the number of estimated locations between observations should improve the approximation because it makes the time discretization finer. This was investigated by simulating from the underlying continuous time model in the special case that reduces to the CTCRW. In this case, the GDCRW is a discretization of the true model. To investigate the effect of the discretization, the ability of the GDCRW to estimate the main movement parameter, *γ* = *γ*_1_ = *γ*_2_, along with the ability to reconstruct the trajectory was evaluated against the CTCRW.

To evaluate the GDCRW against the CTCRW, 200 trajectories with 250 observations were simulated from the continuous time model defined by equations (1) and (2) with *θ* = 0 and diagonal *S*; that is, with the CTCRW as the true model. A total of six models were fitted for each simulated dataset. The CTCRW and the GDCRW with estimated locations at the time of observations were fitted. Further, the GDCRW with additional estimated locations every 8, 4, 2, and 1 time unit were fitted. The models with additional estimated locations were denoted GDCRW_8_, GDCRW_4_, GDCRW_2_, and GDCRW_1_, respectively.

For each of the 250 simulated locations, observations were simulated from a bivariate normal with covariance ${0.1}^{2}{I}_{2}$. The processes were simulated with *γ* = *γ*_1_ = *γ*_2_ = 0.9, *μ* = 0, and ${S}_{11}={S}_{22}={e}^{-2}\approx 0.135$. For simplicity, the model was fitted with *γ* = *γ*_1_ = *γ*_2_, *θ* = 0, *μ* = 0, and ${S}_{12}={S}_{21}\mathrm{=0}$.

The CTCRW models both the velocity and location giving a total of 1000 latent variables: 4 for each location. In contrast, the GDCRW only has 500 latent variables: 2 for each location. The mean of the time step distribution used in the simulation study was approximately 1.99, giving an average length of a simulated trajectory of 497.5 time units. Therefore, the GDCRW_8_ would include 63 additional location estimates on average, corresponding to a total of 626 latent variables. Likewise, the GDCRW_4_, GDCRW_2_, and GDCRW_1_ would on average include 125, 249, and 498 additional locations, respectively, corresponding to 750, 998, and 1496 latent variables.

**Figure 1:**
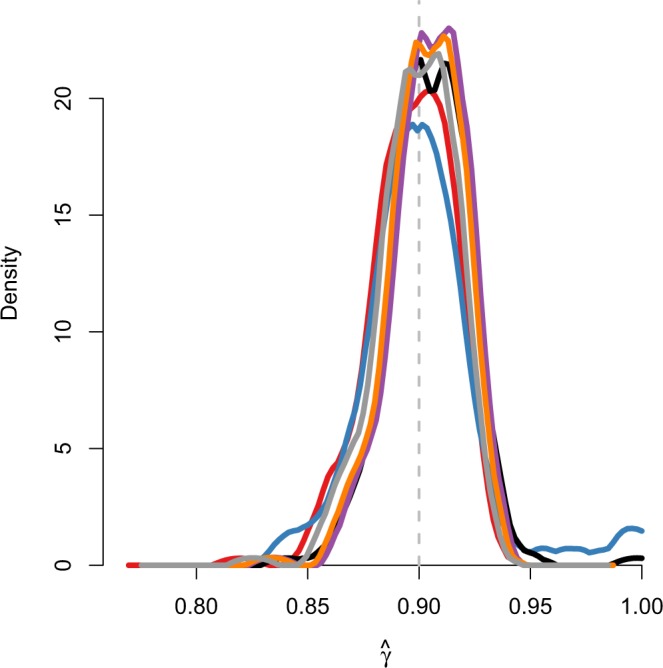
Density of autocorrelation parameter, *γ*, estimates in the simulation study for the models: CTCRW (red line), GDCRW (blue line), GDCRW_8_ (black line), GDCRW_4_ (purple line), GDCRW_2_ (orange line), and GDCRW_1_ (grey line). A GDCRW_*i*_ model includes additional estimated locations every *i* time unit. Grey dashed line indicates the true parameter value.

Regardless of the different number of latent variables, all six estimation models (CTCRW, GDCRW, and the GDCRW with additional location estimates every 8, 4, 2, and 1 time unit) provided *γ* estimates close to the true value (Fig. 1); however, the standard deviation of the estimates was higher for the GDCRW than for the five other models. For the true continuous time model, the CTCRW, the average of the 200 estimates (standard deviation) was 0.897 (0.018), whereas it was 0.901 (0.028) for the discrete time approximation with estimated locations only at the time of the observations, the GDCRW. When additional estimated locations were included every 8, 4, 2, and 1 time unit, the average of the estimated *γ* s were 0.905 (0.018), 0.905 (0.016), 0.903 (0.016), and 0.899 (0.017), respectively.

**Figure 2:**
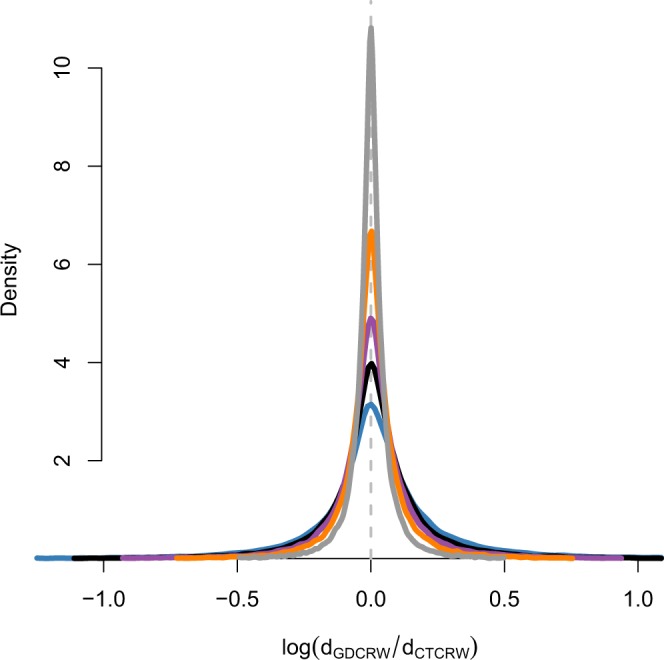
Density of distance between estimated locations and true simulated locations in the simulation study for the models: GDCRW (blue line), GDCRW_8_ (black line), GDCRW_4_ (purple line), GDCRW_2_ (orange line), and GDCRW_1_ (grey line) relative to the CTCRW. The log-ratio is shown. A value of zero (grey dashed line) indicates the median distance is the same as for the CTCRW. The horizontal axis is cut to show 99% of the data. The full range is (−5.317; 4.141). Values from all simulated trajectories are combined.

Besides the ability to re-obtain the true parameters, the GDCRW models with different numbers of estimated locations were evaluated on the distance between the estimated locations and the true simulated locations (Fig. 2). To evaluate the models, this distance was calculated for each location for each track for the GDCRW, GDCRW_8_, GDCRW_4_, GDCRW_2_, and GDCRW_1_, and divided by the distance obtained for the CTCRW to get a relative distance. Note that since the coordinate system of the simulation study is arbitrary, the distances are only meaningful relative to each other. Whereas the models performed similarly in estimating *γ*, the CTCRW performed slightly better than the other models in obtaining precise location estimates; however, the GDCRW models improved when additional locations were estimated, as expected. For the GDCRW, 46% of the estimated locations were closer to the true location than the corresponding estimated location from the CTCRW. For the GDCRW_8_, 46.9% of the estimated locations were closer to the true location than the CTCRW, while it was 47.9, 48.5, and 49.6%, respectively, for the GDCRW_4_, GDCRW_2_, and GDCRW_1_. Consequently, the median relative distances where 1.0125 (GDCRW), 1.0075 (GDCRW_8_), 1.0043 (GDCRW_4_), 1.0021 (GDCRW_2_), and 1.0003 (GDCRW_1_). Further, the range of relative distances narrowed when the number of estimated locations increased. When no additional estimated locations were included, the length of the range on log-scale was 9.458 (−5.317; 4.141). Based on these 200 simulations, the range gradually narrowed for the GDCRW_8_ (8.894; min: −5.024; max: 3.87), GDCRW_4_ (7.711; min: −4.069; max: 3.642), GDCRW_2_ (6.746; min: −3.252; max: 3.494), and GDCRW_1_ (6.655; min: −2.938; max: 3.718).

This simulation study evaluated the effect of the discretization used by the GDCRW when the true movement was continuous in time. Even when assuming constant velocity between observations, the GDCRW performed well compared to the CTCRW from which the data was simulated. Although the GDCRW with estimated locations at the time of observations performed well, performance could be improved by including additional estimated locations between observations. Including additional estimated locations made the time discretization finer, thereby allowing the velocity to change between observations and improving the Euler-Maruyama approximation. For a sufficient number of additional estimated locations, the discretized movement model would resemble the true continuous time model.

### Comparing the GDCRW and the DCRW

Above it was established that the DCRW was a special case of the GDCRW. One of the ways the DCRW was generalized was that the GDCRW allowed estimated locations at arbitrary time points. In contrast, the DCRW only allowed regular time steps in discretizing the underlying SDE. In this simulation study the effect of the choice of discretization on parameter estimates and trajectory reconstruction was investigated. Trajectories were simulated from the underlying continuous time model in the special case that would reduce to the DCRW if the time steps were regular. The comparison consists of three parts. The first two parts compare how the DCRW and GDCRW are able to estimate parameters of the continuous time movement. The comparisons are made for a tortuous and a persistent movement, respectively. The final part compares how the DCRW and GDCRW are able to reconstruct the true trajectories, and how they are influenced by measurement error.

Datasets were simulated from two behavioural scenarios: a tortuous movement scenario with low autocorrelation and non-zero mean turning angle, and a persistent movement scenario with high autocorrelation and a mean turning angle equal to zero. The scenarios were selected to reflect the typical behavioural states the DCRW is used for (e.g^13,21^.). For each scenario, 200 trajectories with 250 observations were simulated. Measurements were simulated from a normal distribution with covariance ${{\rm{\Sigma }}}_{{\rm{o}}}={0.1}^{2}{I}_{2}$.

Both the DCRW and the GDCRW are discretizations of the true model. As a result, the models are expected to perform better when the number of estimated locations is increased, since increasing the number of estimated locations makes the time discretization finer. For each trajectory, a total of 10 models were fitted. Both the GDCRW and the DCRW were fitted with $N=\mathrm{250,500,750,1000,}$ and 1250 estimated locations. In the DCRW, the estimated locations were included at regular time intervals. As mentioned above, observations were, on average, 1.99 time units apart. Consequently, the average trajectory length was approximately 497.5 time units. Therefore, the number of estimated locations (*N*) corresponds to estimated locations every 1.99, 1, 0.66, 0.5, and 0.4 time unit, respectively. In the GDCRW, 250 estimated locations were aligned with the observations. Remaining estimated locations were included recursively in the middle of the longest time step. For simplicity, the DCRW models were fitted with *S*_11_ = *S*_22_ and *S*_12_ = *S*_21_ = 0. The GDCRW models were fitted with *γ* = *γ*_1_ = *γ*_2_, *μ* = 0, *S*_11_ = *S*_22_, and *S*_12_ = *S*_21_ = 0 to reduce to the same parameters as the DCRW models.

#### Persistent movement

The first scenario emulated a persistent movement with *θ* = 0 and ${\gamma }_{1}={\gamma }_{2}\mathrm{=0.9}$. The movement was simulated with diagonal *S* such that ${S}_{11}={S}_{22}={e}^{-2}$.

**Figure 3:**
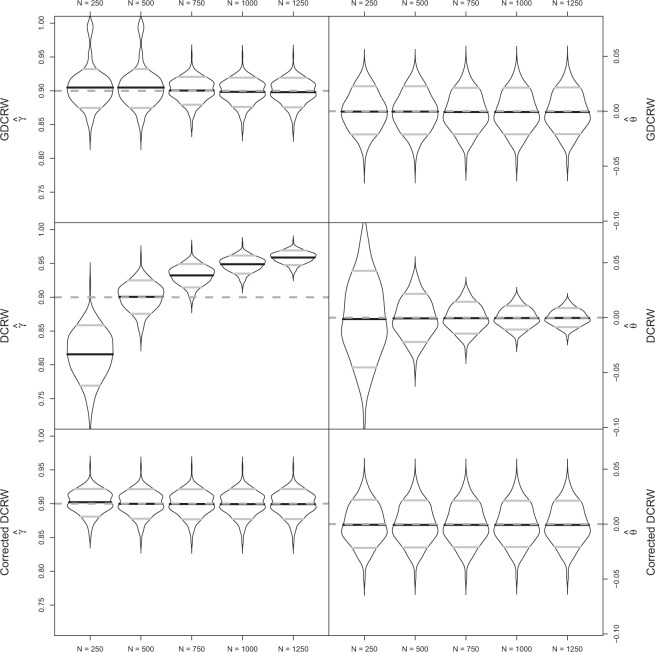
Violin plots of autocorrelation, *γ*, and rotation, *θ*, parameter estimates in the persistent movement simulations for the GDCRW, DCRW, and the DCRW corrected by equation (4) with $N=\mathrm{250,500,750,1000,1250}$ estimated locations and 250 observations. The additional estimated locations were added during the fitting process to make the time discretization of the models finer. Grey dashed lines indicate true parameter values. In the violin plot, black lines indicate the mean of estimates and grey lines indicate 10 and 90% quantiles of estimates.

In this movement scenario, the GDCRW and the DCRW corrected by equation (4) provided estimates close to the true values for both parameters for all five number of estimated locations (Fig. 3). For the GDCRW, the average *γ* estimates ranged from 0.898 to 0.905, and the average *θ* estimates ranged between −0.001 and 0. Likewise, the average DCRW estimates corrected by equation (4) ranged from 0.899 to 0.902 for *γ*, and from −0.001 to −0.001 for *θ*. Unlike for the GDCRW, the estimates obtained directly from the DCRW were not close to the true values. For the DCRW models, the average *γ* estimates increased with the number of estimated locations from 0.815 to 0.959. The average *θ* estimates were all close to zero (min: −0.001; max: 0.000); however, their standard deviation decreased from 0.035 to 0.007 as the number of estimated locations increased.

Similar to the comparison with the CTCRW, the GDCRW performed well for this persistent movement simulation study. For the rotation parameter, *θ*, the estimates barely changed by including additional location estimates, whereas the standard deviation decreased slightly by increasing the number of estimated locations. Likewise, when the DCRW parameters were corrected by equation (4), the DCRW performed well for all five number of location estimates. When the movement was persistent, changes in velocity between observations were small; therefore, few estimated locations were needed for the discretized movement to resemble the true continuous time trajectory.

#### Tortuous movement

The second scenario mimicked a more tortuous movement. Movement was simulated with $\theta =\frac{\pi }{3}\approx 1.05$, *μ* = 0, and ${\gamma }_{1}={\gamma }_{2}=0.6$. Similar to the first scenario, the movement was simulated with ${S}_{11}={S}_{22}={e}^{-2}$.

**Figure 4:**
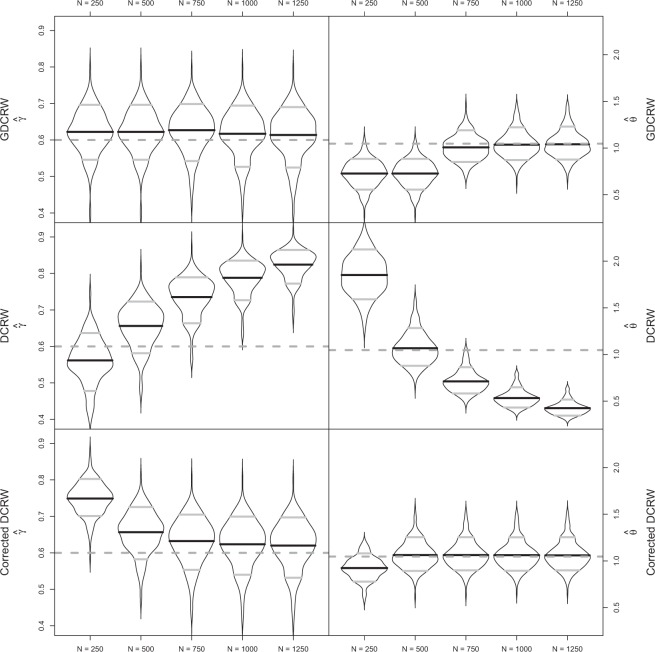
Violin plots of autocorrelation, *γ*, and rotation, *θ*, parameter estimates in the tortuous movement simulations for the GDCRW, DCRW, and the DCRW corrected by equation (4) with $N=\mathrm{250,500,750,1000,1250}$ estimated locations and 250 observations. The additional estimated locations were added during the fitting process to make the time discretization of the models finer. Grey dashed lines indicate true parameter values. In the violin plot, black lines indicate the mean of estimates and grey lines indicate 10 and 90% quantiles of estimates.

The GDCRW provided *γ* estimates close to the true value for all five numbers of estimated locations with a slight tendency to overestimate the value (Fig. 4). The average *γ* estimates (standard deviation) were 0.622 (0.059), 0.622 (0.059), 0.627 (0.061), 0.617 (0.064), and 0.614 (0.066) for the GDCRW with $N=\mathrm{250,500,750,1000,}$ and 1250 estimated locations, respectively. A difference was, however, seen for the *θ* estimates. The GDCRW with 250 and 500 estimated locations provided downwards biased estimates of *θ*. Similarly to the persistent movement scenario, the estimates obtained directly from the DCRW were not close to the true values. For these models, the average *γ* estimates increased from 0.562 to 0.824 with the number of estimated locations, while the average *θ* estimates decreased from 1.851 to 0.426, illustrating the timescale dependence on the parameter estimates. After correcting the DCRW parameter estimates by equation (4), their values came closer to the true parameter value as the number of estimated locations increased. The average *γ* estimates decreased from 0.749 to 0.62, while the average *θ* estimates increased from 0.924 to 1.06.

Unlike the two previous simulation studies, additional estimated locations were necessary for the tortuous movement scenario. For both the GDCRW and the DCRW corrected by equation (4), estimates of the rotation parameter, *θ*, improved by including additional estimated location. The autocorrelation parameter was estimated accurately by the GDCRW for all five number of estimated locations. For the DCRW corrected by equation (4), the estimation improved with the number of estimated locations.

When the movement was tortuous, changes in velocity between observations were larger and more frequent than for a persistent movement. Consequently, more estimated locations were needed for the GDCRW to resemble the true continuous time movement.

From these comparisons with the DCRW, it was evident that parameter estimates of the DCRW depended on the time between estimated locations. When the time between estimated locations decreased, the autocorrelation parameter, *γ*, of the DCRW would increase, while a non-zero turning angle, *θ* ≠ 0, would decrease. While the parameter estimates could be corrected by equation (4), an advantage of the GDCRW is that the parameter estimates are independent of the time scale.

#### Effect of measurement error on the choice of time steps

In the final simulation study, the GDCRW and DCRW were compared once more. While the previous comparison focused on parameter estimates, this simulation study compared the ability to accurately obtain location estimates for different ratios of process variability and measurement variability. Again, 200 trajectories with 250 observations were simulated from two behavioural scenarios using the same procedure as for the two previous simulation studies. The same movement parameters as in the previous simulation study were used, and the GDCRW and DCRW with 250 estimated locations were fitted in the same manner as before; however, the observations were simulated with covariances ${e}^{-2\cdot 6}{I}_{2},{e}^{-2\cdot 5.5}{I}_{2},\ldots ,{e}^{2\cdot 1.5}{I}_{2},{e}^{2\cdot 2}{I}_{2}$, respectively. For each estimated location, the distance to the true location was calculated and compared between the GDCRW and the DCRW. Comparisons were made by dividing GDCRW distances with the corresponding DCRW distances to obtain relative distances. The DCRW locations were interpolated linearly to get values at the time of observations.

**Figure 5:**
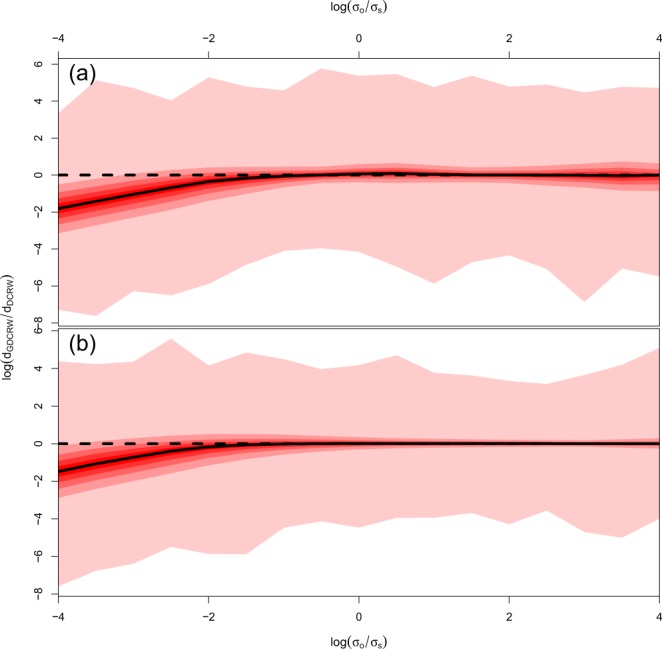
Distribution of distances between estimated locations and true simulated locations for the GDCRW relative to the DCRW in the tortuous (**a**) and persistent (**b**) simulated movement scenarios as a function of the ratio between observational standard deviation (*σ*_*o*_) and movement standard deviation (*σ*_*s*_). The red bands show the full range, 0.1–0.9, 0.2–0.8, 0.3–0.7, and 0.4–0.6 quantile intervals. The median is shown by the full black line. Values below 0 (black dashed line) indicates that the estimated location in the GDCRW was closer to the true value than for the DCRW.

When the measurement standard deviation was low, the GDCRW performed notably better than the DCRW (Fig. 5). In both scenarios, 90% of the simulated locations had a relative distance less than 1; that is, the locations were better estimated by the GDCRW than the DCRW. As the measurement standard deviation increased relative to the movement standard deviation, the median relative distance tended to one, suggesting that the GDCRW and the DCRW returned similar location estimates.

When measurement error was low, and the number of estimated locations were the same as the number of locations, the GDCRW was better at reconstructing the true trajectories than the DCRW. Hence, it was better to have the estimated locations at the time of the observations than regularly spaced in time, because when the estimated locations were regularly spaced in time, several observations could be related to the same estimated locations. Consequently, the estimated location must provide a reasonable compromise for all related observations which was a disadvantage compared to the GDCRW when the measurement error was small. As the measurement error of each observation increases, the uncertainty of the GDCRW location estimate also increases and the two methods give similar results.

## Case study

To illustrate its practical applicability, the GDCRW model was fitted to the subadult ringed seal *Pusa hispida* data of Albertsen *et al*.^7^ in this case study. Albertsen *et al*.^7^ fitted both the CTCRW and DCRW to the data. The dataset was chosen to illustrate the movement model when substantial measurement errors are present. The ringed seal data consists of *N* = 3583 locations collected by the Argos satellite system, which is known to have substantial non-Gaussian measurement errors. Three observations with location class Z were excluded. In the first period of the track, the seal was moving north-west, followed by two periods with restricted space use close to land. Consequently, Thygesen *et al*.^27^ found indications that the CTCRW was not sufficient, but a non-constant drift parameter was needed. To account for this behaviour, the mean velocity parameters of the GDCRW were set to be location dependent:

$${\rm{\Theta }}=(\begin{array}{cc}-\mathrm{log}\,{\gamma }_{lat} & \theta \\ -\theta  & -\,\mathrm{log}\,{\gamma }_{lon}\end{array})$$

$${X}_{{t}_{i}}={X}_{{t}_{i-1}}+{{\rm{\Delta }}}_{{t}_{i}}{e}^{-{{\rm{\Theta }}{\rm{\Delta }}}_{{t}_{i-1}}}({X}_{{t}_{i-1}}-{X}_{{t}_{i-2}})/{{\rm{\Delta }}}_{{t}_{i-1}}+{{\rm{\Delta }}}_{{t}_{i}}({I}_{2}-{e}^{-{{\rm{\Theta }}{\rm{\Delta }}}_{{t}_{i-1}}})\mu ({X}_{{t}_{i-1}})+{{\rm{\Delta }}}_{{t}_{i}}{\varepsilon }_{{t}_{i}}$$

The bivariate mean velocity field $\mu ({X}_{t})=({\mu }_{lat}({X}_{t}),{\mu }_{lon}({X}_{t}{))}^{T}$ was modelled through a 15 × 15 grid of random effects. Note that like Johnson *et al*.^20^, the argosTrack package used for inference has latitude as the first coordinate^26^. The order of the coordinates only affects the sign of *θ*. Each grid point was related to two random effects: one for the mean latitudinal velocity, *μ*_1_, and one for the mean longitudinal velocity, *μ*_2_. Between the grid points, the random effect values were interpolated by a search tree approximation to local polynomial regression^28^. In this case study, the number of random effects to construct the grids were selected as a trade-off between computational cost and flexibility. Locations of the movement process were estimated at every observation and every hour from the first observation, giving a total of 7459 location estimates each with two coordinates. The number of additional estimated locations was selected to include as many as possible while limiting the computational cost. Following Albertsen *et al*.^7^, the measurements were modelled by a bivariate t-distribution with scale matrices and degrees of freedom depending on the location class of the observations. The model was fitted to the data by maximum likelihood through the Laplace approximation using TMB and argosTrack. Estimating the mean velocity field in TMB was time and memory consuming; therefore, the model was run on a high performance computing cluster with 128 GB memory.

Having a location dependent drift in the model, the animal movement may be related to available resources or hydrographic variables. The model cannot be estimated using the DCRW since the DCRW does not include a drift parameter. Furthermore, the model cannot easily be estimated using CTCRW without assuming constant velocity between estimated locations, in which case the CTCRW is a special case of the GDCRW (see gdcrwVSctcrw).

**Figure 6:**
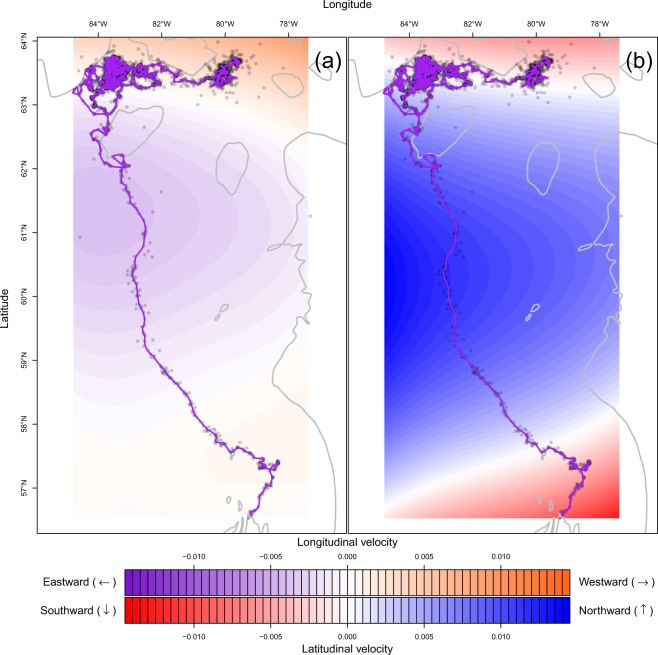
Mean longitudinal (**a**) and latitudinal (**b**) velocity field of the subadult ringed seal track with land (outlined by grey lines), Argos satellite observations (dark grey dots), and fitted most probable track given all observations (purple line). In the longitudinal field, red values indicate an eastward attraction, while blue values indicate a westward attraction. In the latitudinal field, red values indicate a northward attraction, while blue values indicate a southward attraction.

The fitted model resulted in small estimated longitudinal mean velocity field values (Fig. 6). On the contrary, the latitudinal mean velocity field was estimated to have a southward drift when the seal was south of the 57.5°N parallel. Despite the southward drift, the seal then entered a large area of northward drift between 57.5°N and 63.5°N. North of the 63.5°N parallel, the latitudinal mean velocity field was estimated to have a southward attraction, keeping the seal close to the 63.5°N parallel.

Further, the model estimated an overall mean turning angle, *θ*. This parameter was estimated to be significantly different from zero (Table 2), albeit close, whereas both the latitudinal and longitudinal autocorrelation parameters were estimated to be close to 0.56. Combined, this indicated a slightly tortuous movement; slightly circular, with neither high nor low autocorrelation when the drift was zero. When the drift in either direction was not zero, the movement was directed, and deviations from the drift were slightly tortuous. The estimated autocorrelation was lowered by including the velocity field in the model. Albertsen *et al*.^7^ estimated the autocorrelation in the CTCRW to be 0.65. Whereas the CTCRW explained directed movement by a high autocorrelation, this case study explained directed movement by a drift related to the seal’s position. Naturally, the drift lowers the remaining autocorrelation in the trajectory. All movement parameter estimates and their standard errors are seen in Table 2.

**Table 2:** Estimated movement parameters in the ringed seal case study.

	Estimate	Standard Error
*γ*_*lat*_	0.561	0.018
*γ*_*lon*_	0.569	0.024
*θ*	0.037	0.007
${{\rm{\Sigma }}}_{lat,lon}/\sqrt{{{\rm{\Sigma }}}_{lat,lat}{{\rm{\Sigma }}}_{lon,lon}}$	−0.008	0.010
${\mathrm{log}{\rm{\Sigma }}}_{lat,lat}$	−8.836	0.058
${\mathrm{log}{\rm{\Sigma }}}_{lon,lon}$	−7.303	0.106

## Discussion

The GDCRW introduced here generalizes the DCRW movement model of Jonsen *et al*.^13^ in three ways. Firstly, whereas the DCRW handles irregular observations by linear interpolation in the observational model, the GDCRW allows modelling irregular observations directly in the movement process. Through this construction, time-scale corrections of the DCRW parameters were found by considering a time regular GDCRW. Secondly, the GDCRW generalizes the DCRW by allowing different autocorrelation parameters in the two coordinates. Hence, when the rotation parameter is zero, the GDCRW resembles the integrated velocity model of the CTCRW of Johnson *et al*.^20^. Like the CTCRW and DCRW, the GDCRW includes the random walk as a limiting case. When the autocorrelation parameter approaches zero, the movement model approaches that of a random walk, which has been used in several studies, in particular when the data includes only daily observations (e.g.^29,30^). Thirdly, the GDCRW extends the DCRW by including a drift term. Because the GDCRW is more general, it is also computationally more demanding to fit the model to data (see Supplementary Material S4). While the GDCRW has fewer latent variables than the CTCRW, and the same number as the DCRW, the transition equation includes matrix exponentials, which are computationally demanding. In the implementation used here, the matrix exponentials are calculated in each time step^26^; however, estimation can, in general, be made faster by reusing calculations.

When the true animal movement is continuous in time, the GDCRW is a discrete time approximation. The quality of the approximation can be improved by including additional estimated locations between observations. The additional estimated locations make the time discretization finer, effectively allowing the discrete time movement to change several times between observations, similar to the continuous time movement. Likewise, the DCRW provides a discrete time approximation; but, with regular time steps. In the simulation studies, it was shown that it is generally better to place estimated locations at the time of observations than regularly throughout the track. This was particularly true when measurement error was low. Nonetheless, when computational power is limited, regular time steps can be used to decrease the number of estimated locations to fewer than the number of observations. Both the GDCRW and DCRW can be improved as approximations to continuous time movement by adding additional estimated locations between observations. The GDCRW, and the DCRW as a special case, is an Euler-Maruyama approximation of the continuous time movement defined by equations (1) and (2). Increasing the number of estimated locations will make the time discretization of the approximation finer, thereby improving it. While more estimated locations are better, the number used in practice will often be limited by the increasing computational complexity of the estimation method. Further, the number needed depends on the type of movement analysed. For persistent movement trajectories with few changes, few additional estimated locations are needed. For tortuous movement trajectories with rapid changes of velocity and direction, more additional estimated locations are needed for accurate inference. The simulation studies suggest that from a certain point, the advantage of including more estimated locations will be limited. In the comparison with the DCRW, parameter estimates of the GDCRW did not improve much when more than 750 locations were estimated for 250 observations (Figs 3 and 4). When the number of additional estimated locations is a concern, the analysis can start by only including estimated locations at the time of observations and gradually increase the number of additional estimated locations until the inference is no longer improved.

Including a drift term in the model is useful for animals moving persistently between areas, such as the ringed seal analysed in the case study. In the case study, the drift term was modelled by a location dependent field. Having location or time dependent parameters in the SDE greatly complicates the calculations to obtain an analytical solution and in turn a fully continuous time model. Therefore, location dependent mean velocities could not easily be implemented in the CTCRW which has previously been used to analyse the track^7,27^. Nonetheless, using the discrete time model, or a discrete time approximation, it is straightforward to extend the movement model with a location dependent drift. Using the same methods, spatial covariates such as sea surface temperature or depth could be included.

While the mean turning angle was estimated to be close to zero using the entire track, this may not be the case if, for instance, a time varying parameter or behavioural switching was included. Changes in behavioural states, ${S}_{{t}_{i}}\in \mathrm{\{1,}\,\mathrm{2,}\ldots ,n\}$, can be modelled by a Markov Chain such that

$$P({S}_{{t}_{i}}=k|{S}_{{t}_{i-1}}=j)={({e}^{Q{{\rm{\Delta }}}_{{t}_{i}}})}_{jk}$$

where *Q* is an *n* × *n* matrix such that ${Q}_{jk}\mathrm{ > 0}$ for $j\ne k$ and ${Q}_{jj}=-{\sum }_{\{k:j\ne k\}}{Q}_{jk}$. The movement model is modified to have different parameters depending on the current behavioural state

$${X}_{{t}_{i}}={X}_{{t}_{i-1}}+{{\rm{\Delta }}}_{{t}_{i}}{e}^{-{{\rm{\Theta }}}_{{S}_{{t}_{i}}}{{\rm{\Delta }}}_{{t}_{i}}}({X}_{{t}_{i-1}}-{X}_{{t}_{i-2}})/{{\rm{\Delta }}}_{{t}_{i-1}}+{{\rm{\Delta }}}_{{t}_{i}}({I}_{2}-{e}^{-{{\rm{\Theta }}}_{{S}_{{t}_{i}}}{{\rm{\Delta }}}_{{t}_{i}}}){\mu }_{{S}_{{t}_{i}}}+{{\rm{\Delta }}}_{{t}_{i}}{\varepsilon }_{{t}_{i}}\mathrm{.}$$

This model generalizes the behavioural switching movement models of Jonsen *et al*.^13^ and Whoriskey *et al*.^21^. It assumes that switching only occurs at the predetermined time points. That is, between two estimated locations, the animal only exhibits one type of behaviour and moves at a constant velocity; however, as it has been shown in both the case study and simulation studies, additional locations can be estimated either at regular time points, or at the time of observations and any time between them. While the extension to include behavioural switching is simple, maximum likelihood estimation is greatly complicated when measurement error is present. When observations are obtained without error, behavioural states and parameters can be estimated using hidden Markov model methods (e.g.^21^).

The GDCRW can be constructed as a discretization of a generalization of the CTCRW. The first simulation study comparing the GDCRW and CTCRW suggests that using the analytical continuous time solution, when it can be found, generally provides better results than a discrete time approximation. Nonetheless, the discrete time approximation provided accurate parameter estimates, even when no additional estimated locations were included to improve the approximation. While the GDCRW was barely outperformed by the CTCRW in estimating true locations when the CTCRW was the true model, the first simulation study illustrated that the performance of the GDCRW could be improved to closely resemble the CTCRW. The performance of the GDCRW could be improved by including additional estimated locations between observations, because the movement process is then discretized on a finer time grid. Therefore, if the only aim is to reconstruct the true trajectory, any of the models perform well. Otherwise, the choice between the models should be based on which parameters are important to estimate.

The second simulation study compared the DCRW and the GDCRW in their ability to re-estimate the movement parameters of the underlying continuous time model. In both the tortuous and persistent movement scenarios, the GDCRW performed well; however, in the tortuous movement scenario, the performance was notably improved by adding additional estimated locations between observations. Further, it became evident that the parameter estimates of the DCRW are highly dependent on the selected time steps between estimated locations. Nevertheless, the estimates could be corrected by equation (4): the GDCRW with regular time steps. This correction provided estimates close to the true values in all cases except in the tortuous movement case with 250 and 500 estimated locations. Clearly, the ability to have time-scale independent movement parameters is a key feature of the GDCRW, regardless of whether it is used with regular or irregular time steps. Time-scale independent movement parameters allow analysis of several animals without using the same time steps. Further, they allow comparing results from previous studies using the DCRW even if different time steps are used.

Besides accuracy of the estimates, the DCRW and GDCRW were compared on their ability to reconstruct the movement tracks. The third simulation study showed that modelling time-irregular data through a time-irregular movement could increase the accuracy of predicted locations. This is consistent with previous findings comparing the DCRW and CTCRW, which showed that the continuous time model performed better if both movement models were combined with t-distributed measurement errors^7^. Overall, using the GDCRW with irregular time steps performed well in both scenarios compared to the DCRW. For small measurement errors, the irregular time steps outperformed the regular time steps with interpolation. For medium measurement errors, the two approaches performed equally in the persistent movement scenario, whereas the DCRW slightly outperformed the GDCRW in the tortuous movement case. For large measurement errors, the two approaches performed equally in both scenarios. Combined with the previous simulation study, these results suggest that the GDCRW performs well compared to the DCRW. In general, it is an advantage to have estimated locations at the time of observations for reconstructing trajectories. However, both models improve as approximations to the same underlying continuous time movement model when the number of estimated locations increases.

In this paper, a new method for dealing with telemetry data made up of time irregular locations was introduced. The model introduced was shown to generalize the DCRW and it was evaluated through simulation studies. Finally, the applicability of the model was illustrated by a case study including real telemetry data with time irregular observations. Directly modelling irregular data eases interpretation and comparison of movement parameters, because they are defined independently of the time between observations. From equation (4), parameters of the DCRW and GDCRW can be transformed to any time scale. However, the simulation study shows that fitting the DCRW with a poorly chosen time step introduces bias. This poses a problem in population and meta studies. With a discrete time model, all animals must be fitted with the same time steps to compare movement parameters. However, if the same time step is not optimal for all animals, bias can be introduced. Using an irregular or continuous time model transforms the parameters to a common time scale, even when the animals are observed, or behave, at different time scales.

## Supplementary information

Supplementary Information

## Data Availability

Data analysed in the case study are available with the R package argosTrack in the Zenodo repository, 10.5281/zenodo.1420418. Code for the case study is available from the supplementary material and from GitHub, https://github.com/calbertsen/argosTrack/tree/c3a08ab/argosTrack/inst/examples/gdcrw_velocityfield.

## References

[1] Hussey, N. E. *et al*. Aquatic animal telemetry: A panoramic window into the underwater world. *Science* **348**, 10.1126/science.1255642 (2015). 10.1126/science.1255642 26068859

[2] Costa DP (2010). Accuracy of ARGOS locations of pinnipeds at-sea estimated using fastloc GPS. PLoS One.

[3] Fournier DA (2012). Ad model builder: using automatic differentiation for statistical inference of highly parameterized complex nonlinear models. Optim. Methods Softw..

[4] Kristensen K, Nielsen A, Berg C, Skaug H, Bell B (2016). Tmb: Automatic differentiation and laplace approximation. J. Stat. Softw..

[5] Auger-Méthé M (2016). State-space models’ dirty little secrets: even simple linear gaussian models can have parameter and state estimation problems. Sci. Reports.

[6] Auger-Méthé M (2017). Spatiotemporal modelling of marine movement data using template model builder. Mar. Ecol. Prog. Ser..

[7] Albertsen CM, Whoriskey K, Yurkowski D, Nielsen A, Flemming J (2015). Fast fitting of non-gaussian state-space models to animal movement data via template model builder. Ecology.

[8] Chambault, P. *et al*. Sea surface temperature predicts the movements of an arctic cetacean: the bowhead whale. *Sci. Reports* **8**, 10.1038/s41598-018-27966-1 (2018). 10.1038/s41598-018-27966-1 PMC6018504 29942009

[9] Winton M (2018). Estimating the distribution and relative density of satellite-tagged loggerhead sea turtles using geostatistical mixed effects models. Mar. Ecol. Prog. Ser..

[10] Pedersen MW, Patterson TA, Thygesen UH, Madsen H (2011). Estimating animal behavior and residency from movement data. Oikos.

[11] McClintock BT, Johnson DS, Hooten MB, Ver Hoef JM, Morales JM (2014). When to be discrete: the importance of time formulation in understanding animal movement. Mov. Ecol..

[12] Morales JM, Haydon DT, Frair J, Holsinger KE, Fryxell JM (2004). Extracting more out of relocation data: Building movement models as mixtures of random walks. Ecology.

[13] Jonsen ID, Flemming JM, Myers RA (2005). Robust state–space modeling of animal movement data. Ecology.

[14] Gurarie E, Andrews RD, Laidre KL (2009). A novel method for identifying behavioural changes in animal movement data. Ecol. Lett..

[15] Tracey JA, Zhu J, Crooks KR (2010). Modeling and inference of animal movement using artificial neural networks. Environ. Ecol. Stat..

[16] McClintock BT (2012). A general discrete-time modeling framework for animal movement using multistate random walks. Ecol. Monogr..

[17] Michelot T, Langrock R, Patterson T (2016). A. moveHMM: an r package for the statistical modelling of animal movement data using hidden markov models. Methods Ecol. Evol..

[18] Turchin, P. *Quantitative Analysis of Movement: Measuring and Modeling Population Redistribution in Animals and Plants* (Sinauer, Sunderland, MA, U.S.A, 1998).

[19] Jonsen I (2016). Joint estimation over multiple individuals improves behavioural state inference from animal movement data. Sci. Reports.

[20] Johnson DS, London JM, Lea M-A, Durban JW (2008). Continuous-time correlated random walk model for animal telemetry data. Ecology.

[21] Whoriskey K (2017). A hidden markov movement model for rapidly identifying behavioral states from animal tracks. Ecol. Evol..

[22] Gurarie, E. *et al*. Correlated velocity models as a fundamental unit of animal movement: synthesis and applications. *Mov. Ecol*. **5**, 10.1186/s40462-017-0103-3 (2017). 10.1186/s40462-017-0103-3 PMC5424322 28496983

[23] Pedersen AR (1995). A new approach to maximum likelihood estimation for stochastic differential equations based on discrete observations. Scand. J. Stat..

[24] Pedersen AR (1995). Consistency and asymptotic normality of an approximate maximum likelihood estimator for discretely observed diffusion processes. Bernoulli.

[25] R Core Team R: *A Language and Environment for Statistical Computing*. R Foundation for Statistical Computing, Vienna, Austria (2018).

[26] Albertsen, C. M. argosTrack: *Fit Movement Models to Argos Data for Marine Animals*, 10.5281/zenodo.1420418 R package version 1.2.2, https://github.com/calbertsen/argosTrack/tree/v1.2.2 (2018).

[27] Thygesen UH, Albertsen CM, Berg CW, Kristensen K, Nielsen A (2017). Validation of ecological state space models using the laplace approximation. Environ. Ecol. Stat..

[28] Albertsen, C. M. *covafillr*: *Local Polynomial Regression of State Dependent Covariates in State-Space Models*, R package version 0.4.3, https://CRAN.R-project.org/package=covafillr (2018).

[29] Nielsen A, Bigelow KA, Musyl MK, Sibert JR (2006). Improving light-based geolocation by including sea surface temperature. Fish. Oceanogr..

[30] Lam C, Nielsen A, Sibert J (2010). Incorporating sea-surface temperature to the light-based geolocation model TrackIt. Mar. Ecol. Prog. Ser..

