# Supplementary material for: Generalizing the first-difference correlated random walk for marine animal movement data
Source: Sci Rep. 2019 Mar 8;9:4017. doi: 10.1038/s41598-019-40405-z (PMC6408531; doi:10.1038/s41598-019-40405-z)
Supplement: Supplementary file 1 — Supplementary Information [file 41598_2019_40405_MOESM1_ESM.pdf]

Generalizing the first-difference correlated random walk for marine animal movement data

Supplementary material

Christoffer Moesgaard Albertsen

National Institute of Aquatic Resources, Technical University of Denmark, Kemitorvet 201, DK-2800 Kgs. Lyngby, Denmark

cmoe@aqua.dtu.dk

S1: Solving the SDE

To solve the SDE,

$$\mathrm{d}V_t = -\Theta(V_t - \mu)\mathrm{d}t + SdB_t,$$

consider the process

$$Y_t = e^{\Theta t}(V_t - \mu)$$

Itô's formula yields

$$\begin{aligned} dY_t &= \Theta e^{\Theta t} V_t d_t + e^{\Theta t} dV_t \\ &= \Theta e^{\Theta t} V_t d_t + e^{\Theta t}\left(-\Theta(V_t - \mu)d_t + SdB_t\right) \\ &= e^{\Theta t} SdB_t \end{aligned}$$

Hence,

$$e^{\Theta t}(V_t - \mu) = (V_0 - \mu) + \int_0^t e^{\Theta u} SdB_u$$

which implies

$$\begin{aligned} V_t - \mu &= e^{-\Theta t}(V_0 - \mu) + e^{-\Theta t}\int_0^t e^{\Theta s} SdB_s \\ &= e^{-\Theta t}V_0 + \left(I - e^{-\Theta t}\right)\mu + \int_0^t e^{-\Theta(t-s)} SdB_s \end{aligned}$$

Since this is a sum of a deterministic term and an integral of a deterministic function with respect to a Wiener process with Gaussian increments, the distribution is Gaussian. The mean of the increment is

$$\begin{aligned} E(V_t \mid V_0) &= E\left(e^{-\Theta t}V_0 + \left(I - e^{-\Theta t}\right)\mu \mid V_0\right) + E\left(\int_0^t e^{-\Theta(t-s)} SdB_s \mid V_0\right) \\ &= e^{-\Theta t}V_0 + \left(I - e^{-\Theta t}\right)\mu \end{aligned}$$

Using Itô isometry, the variance is

$$
\begin{aligned}
Var(V_t \mid V_0) &= Var\left(e^{-\Theta t}V_0 + \left(I - e^{-\Theta t}\right)\mu + \int_0^t e^{-\Theta(t-s)} S dB_s \mid V_0\right) \\
&= Var\left(\int_0^t e^{-\Theta(t-s)} S dB_s \mid V_0\right) \\
&= Var\left(\int_0^t e^{-\Theta(t-s)} S dB_s\right) \\
&= E\left(\left(\int_0^t e^{-\Theta(t-s)} S dB_s\right)^2\right) - E\left(\int_0^t e^{-\Theta(t-s)} S dB_s\right)^2 \\
&= E\left(\left(\int_0^t e^{-\Theta(t-s)} S dB_s\right)^2\right) \\
&= \int_0^t e^{-\Theta(t-s)} SS^T \left(e^{-\Theta(t-s)}\right)^T ds \\
&= \int_0^t e^{-\Theta(t-s)} \Sigma \left(e^{-\Theta(t-s)}\right)^T ds
\end{aligned}
$$

Now[1],

$$
\begin{aligned}
\text{vec}(Var(V_t \mid V_0)) &= \int_0^t e^{-\Theta(t-s)} \otimes e^{-\Theta(t-s)} \, \text{vec}(\Sigma) ds \\
&= \int_0^t e^{-\Theta \oplus \Theta (t-s)} ds \, \text{vec}(\Sigma) \\
&= (\Theta \oplus \Theta)^{-1} \left(I - e^{-\Theta \oplus \Theta t}\right) \text{vec}(\Sigma)
\end{aligned}
$$

where $\oplus$ denotes the Kronecker sum, $A \oplus B = A \otimes I_B + I_A \otimes B$.

Defining the matrix C such that $\text{vec}(C) = (\Theta \oplus \Theta)^{-1} \text{vec}(\Sigma)$,

$$
\begin{aligned}
\text{vec}(Var(V_t \mid V_0)) &= (\Theta \oplus \Theta)^{-1} \text{vec}(\Sigma) - (\Theta \oplus \Theta)^{-1} e^{-\Theta \oplus \Theta t} \text{vec}(\Sigma) \\
&= (\Theta \oplus \Theta)^{-1} \text{vec}(\Sigma) - e^{-\Theta \oplus \Theta t} (\Theta \oplus \Theta)^{-1} \text{vec}(\Sigma) \\
&= \text{vec}(C) - e^{-\Theta \oplus \Theta t} \text{vec}(C) \\
&= \text{vec}(C) - e^{-\Theta t} \otimes e^{-\Theta t} \text{vec}(C)
\end{aligned}
$$

Hence,

$$
Var(V_t \mid V_0) = C - e^{-\Theta t} C e^{-\Theta^T t}
$$

S2: Likelihood function

The GDCRW model is defined by the model equation

$$
X_{t_i} = X_{t_{i-1}} + \Delta_i \exp(-\Theta \Delta_{i-1})(X_{t_{i-1}} - X_{t_{i-2}})/\Delta_{i-1} + \Delta_i \left(I - \exp(-\Theta \Delta_{i-1})\right)\mu + \Delta_i \varepsilon_{t_i},
$$

where the error terms, ε_{t_i}, are normally distributed with mean zero and variance

$$
Var(\varepsilon_{t_i}) = C - \exp(-\Theta \Delta_i) C \exp(-\Theta^T \Delta_i)
$$

with

$$
\text{vec}(C) = (\Theta \oplus \Theta)^{-1} \text{vec}(\Sigma),
$$

and Σ is a covariance matrix.

Consequently, the transition density of the process, $f(X_{t_i} \mid X_{t_{i-1}}, X_{t_{i-2}})$, is bivariate Gaussian with mean

$$X_{t_{i-1}} + \Delta_i \exp(-\Theta\Delta_{i-1})(X_{t_{i-1}} - X_{t_{i-2}})/\Delta_{i-1} + \Delta_i (I - \exp(-\Theta\Delta_{i-1}))\,\mu$$

and the same variance as ε_{t_i}.

Given a measurement density, $g(Y_{t_i} \mid X_{t_i})$, the joint density of all true and observed locations is

$$h(\mathbf{X},\mathbf{Y}) = f(X_{t_1})g(Y_{t_1} \mid X_{t_1})f(X_{t_2} \mid X_{t_1})g(Y_{t_2} \mid X_{t_2})\prod_{i=3}^{N} f(X_{t_i} \mid X_{t_{i-1}}, X_{t_{i-2}})g(Y_{t_i} \mid X_{t_i}),$$

where $\mathbf{X} = \{X_{t_i}\}_{i\in\{1,\dots,N\}}$, $\mathbf{Y} = \{Y_{t_i}\}_{i\in\{1,\dots,N\}}$ and $f(X_{t_1})$ and $f(X_{t_2} \mid X_{t_1})$ are initial distributions. In the `R` package `argosTrack` (version 1.2.2[2]) used for inference here, $f(X_{t_1})$ is unspecified, corresponding to a flat prior, while $X_{t_2} \mid X_{t_1}$ is Gaussian with mean $X_{t_1} - \mu$ and the same variance structure as the rest of the process. This choice is arbitrary; however, as the length of the trajectory increases, the influence of the initial distributions decrease. Since the true locations are unobserved, the marginal density of the observed locations must be used for inference:

$$h(\mathbf{Y}) = \int_{\mathbb{R}^{\mathbb{N}}} h(\{X_{t_i}\}_{i\in\{1,\dots,N\}}, \{Y_{t_i}\}_{i\in\{1,\dots,N\}})d\mathbf{X}.$$

In general, this integral is intractable. To obtain an approximate marginal density, the Laplace approximation is used. The Laplace approximation replaces the integrand with a Gaussian density with the same mode and curvature at the mode (see e.g.[3]) to obtain a tractable integral. For maximum likelihood inference, the marginal density is considered a function of the unknown parameters. Subsequently, true locations can be estimated (or more accurately predicted) given the observations and parameters by

$$\hat{\mathbf{X}}\,\mathrm{argmax}_{\mathbf{X}}\, h(\mathbf{X},\mathbf{Y})$$

Since the GDCRW model is a discrete time model, although allowing irregular time steps, movement velocity is assumed to be constant between estimated locations. To allow changes in velocity between observations, additional estimated locations can be included. This corresponds to adding pseudo-observations that are censored on the entire $\mathbb{R}^2$ (i.e. completely unobserved) since

$$\int_{\mathbb{R}^2} g(Y_{t_i} \mid X_{t_i})dY_{t_i} = 1.$$

For example, consider a trajectory with observations at time 1, 2, 3, and 4. The joint density of true and observed locations for this trajectory is

$$\begin{aligned} h(\mathbf{X},\mathbf{Y}) = {} & f(X_1)g(Y_1 \mid X_1) \\ & \cdot f(X_2 \mid X_1)g(Y_2 \mid X_2) \\ & \cdot f(X_3 \mid X_3, X_2)g(Y_3 \mid X_3) \\ & \cdot f(X_4 \mid X_4, X_3)g(Y_4 \mid X_4) \end{aligned}$$

Including an additional estimated location at time 3.5, the joint likelihood becomes

$$\begin{aligned} h(\mathbf{X},\mathbf{Y}) = {} & f(X_1)g(Y_1 \mid X_1) \\ & \cdot f(X_2 \mid X_1)g(Y_2 \mid X_2) \\ & \cdot f(X_3 \mid X_3, X_2)g(Y_3 \mid X_3) \\ & \cdot f(X_{3.5} \mid X_3, X_2) \cdot 1 \\ & \cdot f(X_4 \mid X_{3.5}, X_3)g(Y_4 \mid X_4). \end{aligned}$$

This allows movement to change once between the observation at time 3 and the observation at time 4. As the number of additional estimated locations is increased, the GDCRW model will converge to a continuous time process.

S3: Simulation method details

To simulate from the process,

$$\begin{aligned} \mathrm{d}V_t &= -\Theta(V_t - \mu)\mathrm{d}t + SdB_t \\ \mathrm{d}X_t &= V_t\mathrm{d}t \end{aligned}$$

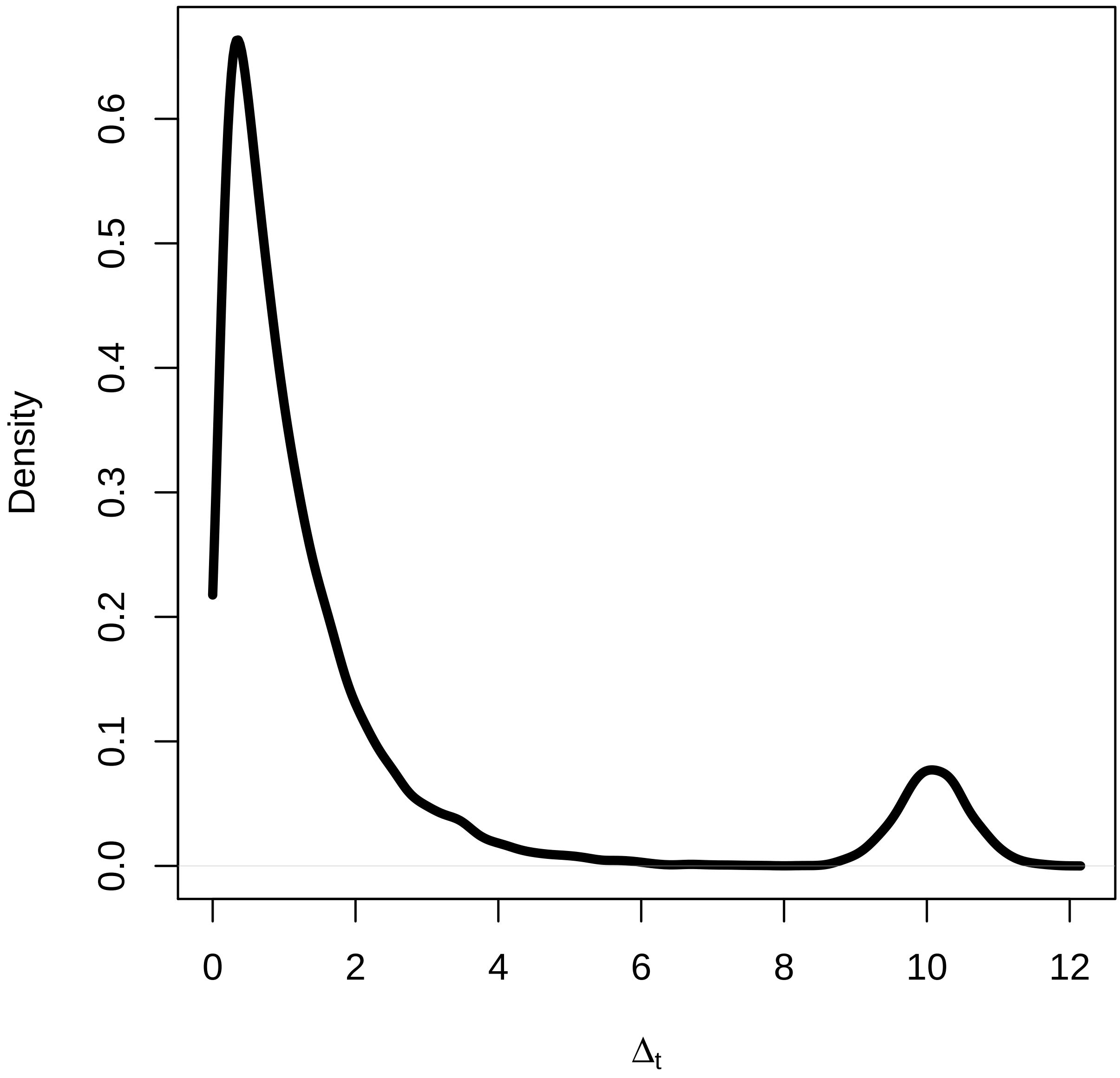

Figure S1. Probability density function used to simulate time steps.

time steps,$\Delta_{t_i} = t_i - t_{i-1}$, between observations where simulated from a mixture of an exponential distribution and a normal distribution. To simulate from the mixture, a uniform random variable, $U \sim unif(0,1)$, an exponentially distributed random variable, $\delta_0 \sim exp(1)$, and a Gaussian random variable, $\delta_1 \sim N(10, 0.5^2)$, were simulated. Then the time step was

$$\Delta_{t_i} = \begin{cases} 0.1 + \delta_0 & U < 0.9 \\ 0.1 + \max(\delta_1, 0.01) & U \geq 0.9 \end{cases}$$

The resulting density function is seen in Figure S1.

Between two time points t_i and t_{i+1}, the processes were simulated using the Euler–Maruyama approximation,

$$V_{s(j+1)} = V_{s(j)} - \Theta(V_n - \mu)\Delta_{s(j+1)} + S\eta_{s(j+1)}$$
$$X_{s(j+1)} = X_{s(j)} + V_{s(j+1)}\Delta_{s(j+1)}$$

with $s(j) = t_i + (n)/199 \cdot \Delta_{t_{i+1}}$, $j = 0, 1, \ldots 199$. Simulated examples can be seen in Figure S2

S4: Simulation study timings

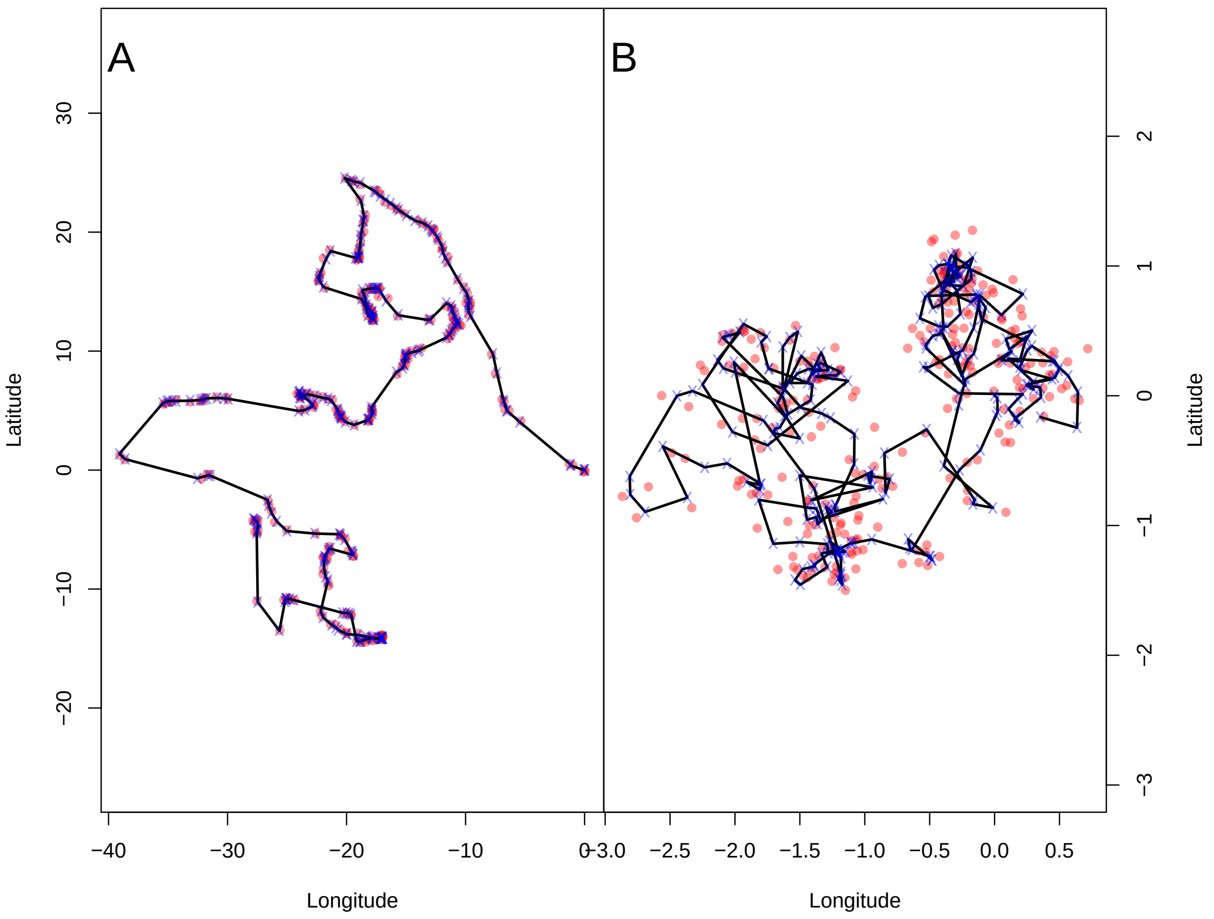

Figure S2. Simulated trajectories (blue crosses connected by black lines) and observations (red points) from the SDE model in the two scenarios: persistent (A) and tortuous (B) movement.

Table 1. Elapsed time in seconds for fitting to simulated data when comparing the CTCRW and GDCRW.

	Minimum	25%	50%	75%	Maximum
CTCRW	9.9	11.9	13.0	14.2	20.0
GDCRW	25.3	30.6	33.0	35.6	43.3
$GDCRW_8$	34.6	43.3	46.8	52.9	72.0
$GDCRW_4$	39.9	54.8	59.9	65.3	88.7
$GDCRW_2$	60.4	74.8	80.2	89.5	106.3
$GDCRW_1$	94.0	112.2	123.2	137.6	213.6

Table 2. Elapsed time in seconds for fitting to simulated data when comparing the DCRW and GDCRW in the tortuous movement scenario.

	Minimum	25%	50%	75%	Maximum
GDCRW(250)	3.2	4.0	4.2	4.5	5.2
GDCRW(500)	3.2	4.0	4.2	4.5	5.4
GDCRW(750)	9.0	10.8	11.4	12.0	13.9
GDCRW(1000)	11.4	14.3	15.1	15.9	18.7
GDCRW(1250)	15.2	17.3	18.5	19.6	22.7
DCRW(250)	0.7	0.9	1.0	1.1	1.6
DCRW(500)	1.1	1.4	1.5	1.7	2.1
DCRW(750)	1.4	2.0	2.1	2.3	2.8
DCRW(1000)	2.0	2.4	2.7	2.9	3.4
DCRW(1250)	2.2	3.1	3.4	3.7	4.7

Table 3. Elapsed time in seconds for fitting to simulated data when comparing the DCRW and GDCRW in the persistent movement scenario.

	Minimum	25%	50%	75%	Maximum
GDCRW(250)	3.6	4.6	4.9	5.2	6.2
GDCRW(500)	3.4	4.5	4.7	5.0	6.1
GDCRW(750)	9.9	11.8	12.5	13.4	15.3
GDCRW(1000)	13.2	15.7	16.6	17.7	21.6
GDCRW(1250)	14.9	19.2	20.5	21.9	26.0
DCRW(250)	0.7	1.0	1.1	1.2	1.5
DCRW(500)	1.1	1.6	1.7	1.8	2.4
DCRW(750)	1.5	2.1	2.3	2.4	3.2
DCRW(1000)	1.8	2.7	2.9	3.1	3.8
DCRW(1250)	2.7	3.4	3.7	4.0	5.3

References

1. Gentle, J. *Matrix Algebra: Theory, Computations, and Applications in Statistics*. Springer Texts in Statistics (Springer, 2007).

2. Albertsen, C. M. *argosTrack: Fit Movement Models to Argos Data for Marine Animals* (2018). DOI 10.5281/zenodo.1420418. R package version 1.2.2. https://github.com/calbertsen/argosTrack/tree/v1.2.2.

3. Kristensen, K., Nielsen, A., Berg, C., Skaug, H. & Bell, B. Tmb: Automatic differentiation and laplace approximation. *J Stat Soft* **70**, 1–21 (2016). DOI 10.18637/jss.v070.i05.

S5: R code for comparing the GDCRW and the CTCRW

```
library(argosTrack)


simDt <- function(n,lambda=1,mu=10,sd=0.5,p=0.9){
    U <- runif(n)
    P <- U < p
    0.1 + P * rexp(n,lambda) +
        (1-P) * sapply(rnorm(n,mu,sd),function(x)max(x,0.01))
}


simModel <- function(nobs = 100,
                     pars = c(0,0,0,0,0,0),
                     varPars = c(0,0),
                     sdMeas = c(0,0),
                     nEul = 200){
    dt0 <- c(0,simDt(nobs-1))
    d0 <- Sys.time() + cumsum(dt0) * 60 * 60
    gamma <- 1/(1+exp(-pars[1:2]))
    phi <- pars[3]
    rho <- 2/(1+exp(-pars[4])) - 1
    mu <- pars[5:6]
    cov <- matrix(c(exp(2*varPars[1]),
                    rho * exp(sum(varPars)),
                    rho * exp(sum(varPars)),
                    exp(2*varPars[2])),2,2)
    S <- t(chol(cov))
    Theta <- matrix(c(-log(gamma[1]),-phi,phi,-log(gamma[2])),2,2)
    oneStep <- function(V0,X0,dt){
        tt <- seq(0,dt,len=nEul)
        dts <- c(NA,diff(tt))
        Xt <- matrix(NA,2,nEul)
        Vt <- matrix(NA,2,nEul)
        Xt[,1] <- X0
        Vt[,1] <- V0
        for(i in 2:nEul){
            Vt[,i] <- Vt[,i-1] - Theta %*% (Vt[,i-1] - mu) * dts[i] +
                S %*% rnorm(2,0,sqrt(dts[i]))
            Xt[,i] <- Xt[,i-1] + dts[i] * Vt[,i-1]
        }
        return(list(X=Xt[,nEul],V=Vt[,nEul]))
    }
    V <- matrix(NA,2,nobs)
    X <- matrix(NA,2,nobs)
    Y <- matrix(NA,2,nobs)
    V[,1] <- 0
    X[,1] <- 0
    Y[1,] <- rnorm(nobs,0,exp(sdMeas[1]))
    Y[2,] <- rnorm(nobs,0,exp(sdMeas[2]))
    for(i in 2:nobs){
        tmp <- oneStep(V[,i-1],X[,i-1],dt0[i])
        V[,i] <- tmp$V
        X[,i] <- tmp$X
    }
    return(list(X=X,Y=Y,dates=d0))
}

for(decay in c(0.9)){
    for(i in 150){
        try({
            nObs <- 250
            pars <- c(qlogis(decay),qlogis(decay),0,0,0,0)
            logSdState <- varPars <- c(-2,-2)
            logSdObs <- sdMeas <- log(c(0.1,0.1))
```

```
        a <- simModel(nObs,pars,varPars,sdMeas)
        obs <- Observation(dates = a$dates,
                           locationclass = rep("GPS",nObs),
                           lon = a$X[2,] + a$Y[2,],
                           lat = a$X[1,] + a$Y[1,])
        anC <- Animal(obs,
                      CTCRW(dates=a$dates,
                            pars= c(0,0,0,0),
                            varPars = logSdState,
                            timeunit="hours"),
                      Measurement(model="n")
                      )
        fitTrack(anC,fixdrift=TRUE,equaldecay=TRUE)
        anI <- Animal(obs,
                      GDCRW(dates=a$dates,timeunit="hours"),
                      Measurement(model="n"))
        fitTrack(anI,fixrotation=TRUE,fixmovecor=TRUE,fixdrift=TRUE,
                 equaldecay=TRUE,equalvar=TRUE)
        dates8 <- unique(sort(c(a$dates,seq(min(a$dates),max(a$dates),
                                            by=8 * 60 * 60))))
        anI8 <- Animal(obs,
                       GDCRW(dates=dates8,timeunit="hours"),
                       Measurement(model="n"))
        fitTrack(anI8,fixrotation=TRUE,fixmovecor=TRUE,fixdrift=TRUE,
                 equaldecay=TRUE,equalvar=TRUE)
        dates4 <- unique(sort(c(a$dates,seq(min(a$dates),max(a$dates),
                                            by=4 * 60 * 60))))
        anI4 <- Animal(obs,
                       GDCRW(dates=dates4),
                       Measurement(model="n"))
        fitTrack(anI4,fixrotation=TRUE,fixmovecor=TRUE,fixdrift=TRUE,
                 equaldecay=TRUE,equalvar=TRUE)
        dates2 <- unique(sort(c(a$dates,seq(min(a$dates),max(a$dates),
                                            by=2 * 60 * 60))))
        anI2 <- Animal(obs,
                       GDCRW(dates=dates2,timeunit="hours"),
                       Measurement(model="n"))
        fitTrack(anI2,fixrotation=TRUE,fixmovecor=TRUE,fixdrift=TRUE,
                 equaldecay=TRUE,equalvar=TRUE)
        dates1 <- unique(sort(c(a$dates,seq(min(a$dates),max(a$dates),
                                            by=1 * 60 * 60))))
        anI1 <- Animal(obs,
                       GDCRW(dates=dates1,timeunit="hours"),
                       Measurement(model="n"))
        fitTrack(anI1,fixrotation=TRUE,fixmovecor=TRUE,fixdrift=TRUE,
                 equaldecay=TRUE,equalvar=TRUE)
        ## Beta
        nam <-
c("True","CTCRW","GDCRW","GDCRW_8","GDCRW_4","GDCRW_2","GDCRW_1")
        tabConv <- c(anC$optim$convergence,
                     anI$optim$convergence,
                     anI8$optim$convergence,
                     anI4$optim$convergence,
                     anI2$optim$convergence,
                     anI1$optim$convergence
                     )
        names(tabConv) <- nam[-1]
        tabBeta <- c(decay,
                     exp(-exp(anC$movement$parameters[1])),
                     exp(--log(1/(1+exp(-anI$movement$parameters[1])))),
                     exp(--log(1/(1+exp(-anI8$movement$parameters[1])))),
                     exp(--log(1/(1+exp(-anI4$movement$parameters[1])))),
                     exp(--log(1/(1+exp(-anI2$movement$parameters[1])))),
                     exp(--log(1/(1+exp(-anI1$movement$parameters[1]))))
```

```
                 )
    names(tabBeta) <- nam
    ## Movement sqrt-Variance
    tabSdState <- rbind(exp(logSdState),
                        exp(anC$movement$varianceParameters),
                        exp(anI$movement$varianceParameters),
                        exp(anI8$movement$varianceParameters),
                        exp(anI4$movement$varianceParameters),
                        exp(anI2$movement$varianceParameters),
                        exp(anI1$movement$varianceParameters)
                        )
    rownames(tabSdState) <- nam
    colnames(tabSdState) <- c("Latitude","Longitude")
    ## Measurement sqrt-Variance
    tabSdObs <- rbind(exp(logSdObs),
                      exp(anC$measurement$logSdObs),
                      exp(anI$measurement$logSdObs),
                      exp(anI8$measurement$logSdObs),
                      exp(anI4$measurement$logSdObs),
                      exp(anI2$measurement$logSdObs),
                      exp(anI1$measurement$logSdObs)
                      )
    rownames(tabSdObs) <- nam
    colnames(tabSdObs) <- c("Latitude","Longitude")
    ## Dist location
    tabDist <- rbind(
    (sqrt(colSums((anC$movement$mu - a$X[1:2,])^2))),
    (sqrt(colSums((anI$movement$mu - a$X[1:2,])^2))),
    (sqrt(colSums((anI8$movement$mu[,which(dates8 %in% a$dates)] -
                   a$X[1:2,])^2))),
    (sqrt(colSums((anI4$movement$mu[,which(dates4 %in% a$dates)] -
                   a$X[1:2,])^2))),
    (sqrt(colSums((anI2$movement$mu[,which(dates2 %in% a$dates)] -
                   a$X[1:2,])^2))),
    (sqrt(colSums((anI1$movement$mu[,which(dates1 %in% a$dates)] -
                   a$X[1:2,])^2)))
    )
    rownames(tabDist) <- nam[-1]
    ## RMSE location
    tabRMSE <- rbind(
        sqrt(rowMeans((anC$movement$mu - a$X[1:2,]) ^ 2)),
        sqrt(rowMeans((anI$movement$mu - a$X[1:2,]) ^ 2)),
        sqrt(rowMeans((anI8$movement$mu[,which(dates8 %in% a$dates)] -
                       a$X[1:2,]) ^ 2)),
        sqrt(rowMeans((anI4$movement$mu[,which(dates4 %in% a$dates)] -
                       a$X[1:2,]) ^ 2)),
        sqrt(rowMeans((anI2$movement$mu[,which(dates2 %in% a$dates)] -
                       a$X[1:2,]) ^ 2)),
        sqrt(rowMeans((anI1$movement$mu[,which(dates1 %in% a$dates)] -
                       a$X[1:2,]) ^ 2))
    )
    rownames(tabRMSE) <- nam[-1]
    colnames(tabRMSE) <- c("Latitude","Longitude")
    tabTime <- rbind(anC$optim$estimation_time,
                     anI$optim$estimation_time,
                     anI8$optim$estimation_time,
                     anI4$optim$estimation_time,
                     anI2$optim$estimation_time,
                     anI1$optim$estimation_time
                     )
    rownames(tabTime) <- nam[-1]
    save(tabConv,tabBeta,tabSdState,tabSdObs,tabDist,tabRMSE, tabTime,
         file=paste0("res_ctcrw_",sub("\\.","p",decay),"_",i,".RData"))
})
```

```
        rm(list=ls()[!(ls() %in% c("i","simDt","simModel","decay"))])
    }
}
```

S6: R code for comparing the GDCRW and the DCRW

```
library(argosTrack)

simDt <- function(n,lambda=1,mu=10,sd=0.5,p=0.9){
    U <- runif(n)
    P <- U < p
    0.1 + P * rexp(n,lambda) + (1-P) * sapply(rnorm(n,mu,sd),
                                          function(x)max(x,0.01))
}

simModel <- function(nobs = 100,
                     pars = c(0,0,0,0,0,0),
                     varPars = c(0,0),
                     sdMeas = c(0,0),
                     nEul = 200){
    dt0 <- c(0,simDt(nobs-1))
    d0 <- Sys.time() + cumsum(dt0) * 60 * 60
    gamma <- 1/(1+exp(-pars[1:2]))
    phi <- pars[3]
    rho <- 2/(1+exp(-pars[4])) - 1
    mu <- pars[5:6]
    cov <- matrix(c(exp(2*varPars[1]),
                    rho * exp(sum(varPars)),
                    rho * exp(sum(varPars)),
                    exp(2*varPars[2])),2,2)
    S <- t(chol(cov))
    Theta <- matrix(c(-log(gamma[1]),-phi,phi,-log(gamma[2])),2,2)
    oneStep <- function(V0,X0,dt){
        tt <- seq(0,dt,len=nEul)
        dts <- c(NA,diff(tt))
        Xt <- matrix(NA,2,nEul)
        Vt <- matrix(NA,2,nEul)
        Xt[,1] <- X0
        Vt[,1] <- V0
        for(i in 2:nEul){
            Vt[,i] <- Vt[,i-1] - Theta %*% (Vt[,i-1] - mu) * dts[i] +
                S %*% rnorm(2,0,sqrt(dts[i]))
            Xt[,i] <- Xt[,i-1] + dts[i] * Vt[,i-1]
        }
        return(list(X=Xt[,nEul],V=Vt[,nEul]))
    }
    V <- matrix(NA,2,nobs)
    X <- matrix(NA,2,nobs)
    Y <- matrix(NA,2,nobs)
    V[,1] <- 0
    X[,1] <- 0
    Y[1,] <- rnorm(nobs,0,exp(sdMeas[1]))
    Y[2,] <- rnorm(nobs,0,exp(sdMeas[2]))
    for(i in 2:nobs){
        tmp <- oneStep(V[,i-1],X[,i-1],dt0[i])
        V[,i] <- tmp$V
        X[,i] <- tmp$X
    }
    return(list(X=X,Y=Y,dates=d0))
}


makeAnimal <- function(a,nDate,ir=TRUE){
    if(ir){
        if(nDate == length(a$dates)){
            dd <- a$dates
        }else{
            dd <- a$dates
            while(length(dd) < nDate){
                dts <- c(0,as.numeric(diff(dd),unit="hours"))
```

```
                ii <- which.max(dts)
                dd <- sort(c(dd,mean(dd[ii+c(-1,0)])))
            }
        }
        mov <- GDCRW(dd,timeunit="hours")
    }else{
        dd <- seq(min(a$dates),max(a$dates),len=nDate)
        mov <- DCRW(dd,timeunit="hours")
    }
    Animal(observation = Observation(lon = a$X[2,] + a$Y[2,],
                                     lat = a$X[1,] + a$Y[1,],
                                     dates = a$dates,
                                     locationclass = rep("GPS",
                                                         length(a$dates))),
           measurement = Measurement(model = "n"),
           movement = mov
           )
}


pars <- rbind(c(qlogis(0.9),qlogis(0.9),0,0,0,0),
              c(qlogis(0.6),qlogis(0.6),pi/3,0,0,0))
rownames(pars) <- c("high","low")
varPars <- c(-2,-2)
sdMeas <- log(c(0.1,0.1))

getDist <- function(a,anim){
    d0 <- a$dates
    d1 <- anim$movement$dates
    mu <- rbind(approx(d1,anim$movement$mu[1,],d0)$y,
                approx(d1,anim$movement$mu[2,],d0)$y)
    dist <- sqrt(colSums((mu - a$X)^2))
    dist
}
getRMSE <- function(a,anim){
    d0 <- a$dates
    d1 <- anim$movement$dates
    mu <- rbind(approx(d1,anim$movement$mu[1,],d0)$y,
                approx(d1,anim$movement$mu[2,],d0)$y)
    RMSE <- sqrt(rowMeans((mu - a$X)^2))
    names(RMSE) <- c("Latitude","Longitude")
    RMSE
}

for(simnr in 1:200)
    for(scenario in 1:nrow(pars)){
        simIsOK <- FALSE
        try({
            a <- simModel(250,pars[scenario,],varPars,sdMeas)
            alb1p0 <- makeAnimal(a,length(a$dates),TRUE)
            fitTrack(alb1p0,fixdrift=TRUE,equaldecay=TRUE,
                     fixmovecor=TRUE,equalvar=TRUE)
            alb2p0 <- makeAnimal(a,length(a$dates)*1,TRUE)
            fitTrack(alb2p0,fixdrift=TRUE,equaldecay=TRUE,
                     fixmovecor=TRUE,equalvar=TRUE)
            alb3p0 <- makeAnimal(a,length(a$dates)*3,TRUE)
            fitTrack(alb3p0,fixdrift=TRUE,equaldecay=TRUE,
                     fixmovecor=TRUE,equalvar=TRUE)
            alb4p0 <- makeAnimal(a,length(a$dates)*4,TRUE)
            fitTrack(alb4p0,fixdrift=TRUE,equaldecay=TRUE,
                     fixmovecor=TRUE,equalvar=TRUE)
            alb5p0 <- makeAnimal(a,length(a$dates)*5,TRUE)
            fitTrack(alb5p0,fixdrift=TRUE,equaldecay=TRUE,
                     fixmovecor=TRUE,equalvar=TRUE)
```

```
jon0p5 <- makeAnimal(a,length(a$dates)*0.5,FALSE)
fitTrack(jon0p5,fixmovecor=TRUE,equalvar=TRUE)
jon1p0 <- makeAnimal(a,length(a$dates)*1,FALSE)
fitTrack(jon1p0,fixmovecor=TRUE,equalvar=TRUE)
jon2p0 <- makeAnimal(a,length(a$dates)*2,FALSE)
fitTrack(jon2p0,fixmovecor=TRUE,equalvar=TRUE)
jon3p0 <- makeAnimal(a,length(a$dates)*3,FALSE)
fitTrack(jon3p0,fixmovecor=TRUE,equalvar=TRUE)
jon4p0 <- makeAnimal(a,length(a$dates)*4,FALSE)
fitTrack(jon4p0,fixmovecor=TRUE,equalvar=TRUE)
jon5p0 <- makeAnimal(a,length(a$dates)*5,FALSE)
fitTrack(jon5p0,fixmovecor=TRUE,equalvar=TRUE)

gdcrwRMSE <- rbind(getRMSE(a,alb1p0),
                   getRMSE(a,alb2p0),
                   getRMSE(a,alb3p0),
                   getRMSE(a,alb4p0),
                   getRMSE(a,alb5p0)
                   )

gdcrwDist <- rbind(getDist(a,alb1p0),
                   getDist(a,alb2p0),
                   getDist(a,alb3p0),
                   getDist(a,alb4p0),
                   getDist(a,alb5p0)
                   )

dcrwRMSE <- rbind(getRMSE(a,jon0p5),
                  getRMSE(a,jon1p0),
                  getRMSE(a,jon2p0),
                  getRMSE(a,jon3p0),
                  getRMSE(a,jon4p0),
                  getRMSE(a,jon5p0))

dcrwDist <- rbind(getDist(a,jon0p5),
                  getDist(a,jon1p0),
                  getDist(a,jon2p0),
                  getDist(a,jon3p0),
                  getDist(a,jon4p0),
                  getDist(a,jon5p0))

nDates <- length(a$dates) * c(0.5,1:5)
conv <- c(alb1p0$optim$convergence,
          alb2p0$optim$convergence,
          alb3p0$optim$convergence,
          alb4p0$optim$convergence,
          alb5p0$optim$convergence)
truePars <- c(pars,varPars)
gdcrwPars <- cbind(conv,nDates[nDates/length(a$dates) >= 1],1,
                   rbind(
                       c(alb1p0$movement$parameters,
                         alb1p0$movement$varianceParameters),
                       c(alb2p0$movement$parameters,
                         alb2p0$movement$varianceParameters),
                       c(alb3p0$movement$parameters,
                         alb3p0$movement$varianceParameters),
                       c(alb4p0$movement$parameters,
                         alb4p0$movement$varianceParameters),
                       c(alb5p0$movement$parameters,
                         alb5p0$movement$varianceParameters)
                   )
                   )
colnames(gdcrwPars) <- c("conv","nDates","dtScale",
```

```
                                        alb1p0$movement$options$parnames,
                                        alb1p0$movement$options$varparnames)

            dt <- c(as.numeric(diff(jon0p5$movement$dates[1:2]),unit="hours"),
                    as.numeric(diff(jon1p0$movement$dates[1:2]),unit="hours"),
                    as.numeric(diff(jon2p0$movement$dates[1:2]),unit="hours"),
                    as.numeric(diff(jon3p0$movement$dates[1:2]),unit="hours"),
                    as.numeric(diff(jon4p0$movement$dates[1:2]),unit="hours"),
                    as.numeric(diff(jon5p0$movement$dates[1:2]),unit="hours"))
            conv <- c(jon0p5$optim$convergence,
                      jon1p0$optim$convergence,
                      jon2p0$optim$convergence,
                      jon3p0$optim$convergence,
                      jon4p0$optim$convergence,
                      jon5p0$optim$convergence)
            dcrwPars <- cbind(conv,nDates,dt,
                              rbind(c(jon0p5$movement$parameters,
                                      jon0p5$movement$varianceParameters),
                                    c(jon1p0$movement$parameters,
                                      jon1p0$movement$varianceParameters),
                                    c(jon2p0$movement$parameters,
                                      jon2p0$movement$varianceParameters),
                                    c(jon3p0$movement$parameters,
                                      jon3p0$movement$varianceParameters),
                                    c(jon4p0$movement$parameters,
                                      jon4p0$movement$varianceParameters),
                                    c(jon5p0$movement$parameters,
                                      jon5p0$movement$varianceParameters)
                                    ))

            colnames(dcrwPars) <- c("conv","nDates","dtScale",
                                    jon1p0$movement$options$parnames,
                                    jon1p0$movement$options$varparnames)

            gdcrwTime <- rbind(alb1p0$optim$estimation_time,
                               alb2p0$optim$estimation_time,
                               alb3p0$optim$estimation_time,
                               alb4p0$optim$estimation_time,
                               alb5p0$optim$estimation_time)

            dcrwTime <- rbind(jon0p5$optim$estimation_time,
                              jon1p0$optim$estimation_time,
                              jon2p0$optim$estimation_time,
                              jon3p0$optim$estimation_time,
                              jon4p0$optim$estimation_time,
                              jon5p0$optim$estimation_time)



            save(truePars,gdcrwPars,dcrwPars,gdcrwRMSE,dcrwRMSE,
                 gdcrwDist,dcrwDist, gdcrwTime, dcrwTime,
                 file=paste0("res_dcrw_",rownames(pars)
[scenario],"_",simnr,".RData"))
        })
        rm(list=ls()[-which(ls() %in% c("scenario","simnr","simDt",
                                        "simModel","pars","varPars",
                                        "sdMeas","makeAnimal","getRMSE",
                                        "getDist"))])
    }
```

S7: R code for evaluating the effect of measurement error on choice of time steps

```
library(argosTrack)

simDt <- function(n,lambda=1,mu=10,sd=0.5,p=0.9){
    U <- runif(n)
    P <- U < p
    0.1 + P * rexp(n,lambda) + (1-P) * sapply(rnorm(n,mu,sd),
                                              function(x)max(x,0.01))
}

simModel <- function(nobs = 100,
                     pars = c(0,0,0,0,0,0),
                     varPars = c(0,0),
                     sdMeas = c(0,0),
                     nEul = 200){
    dt0 <- c(0,simDt(nobs-1))
    d0 <- Sys.time() + cumsum(dt0) * 60 * 60
    gamma <- 1/(1+exp(-pars[1:2]))
    phi <- pars[3]
    rho <- 2/(1+exp(-pars[4])) - 1
    mu <- pars[5:6]
    cov <- matrix(c(exp(2*varPars[1]),
                    rho * exp(sum(varPars)),
                    rho * exp(sum(varPars)),
                    exp(2*varPars[2])),2,2)
    S <- t(chol(cov))
    Theta <- matrix(c(-log(gamma[1]),-phi,phi,-log(gamma[2])),2,2)
    oneStep <- function(V0,X0,dt){
        tt <- seq(0,dt,len=nEul)
        dts <- c(NA,diff(tt))
        Xt <- matrix(NA,2,nEul)
        Vt <- matrix(NA,2,nEul)
        Xt[,1] <- X0
        Vt[,1] <- V0
        for(i in 2:nEul){
            Vt[,i] <- Vt[,i-1] - Theta %*% (Vt[,i-1] - mu) * dts[i] +
                S %*% rnorm(2,0,sqrt(dts[i]))
            Xt[,i] <- Xt[,i-1] + dts[i] * Vt[,i-1]
        }
        return(list(X=Xt[,nEul],V=Vt[,nEul]))
    }
    V <- matrix(NA,2,nobs)
    X <- matrix(NA,2,nobs)
    Y <- matrix(NA,2,nobs)
    V[,1] <- 0
    X[,1] <- 0
    Y[1,] <- rnorm(nobs,0,exp(sdMeas[1]))
    Y[2,] <- rnorm(nobs,0,exp(sdMeas[2]))
    for(i in 2:nobs){
        tmp <- oneStep(V[,i-1],X[,i-1],dt0[i])
        V[,i] <- tmp$V
        X[,i] <- tmp$X
    }
    return(list(X=X,Y=Y,dates=d0))
}

makeAnimal <- function(a,nDate,ir=TRUE){
    if(ir){
        if(nDate == length(a$dates)){
            dd <- a$dates
        }else{
            dd <- a$dates
            while(length(dd) < nDate){
                dts <- c(0,as.numeric(diff(dd),unit="hours"))
                ii <- which.max(dts)
```

```
                dd <- sort(c(dd,mean(dd[ii+c(-1,0)])))
            }
        }
        mov <- GDCRW(dd,timeunit="hours")
    }else{
        dd <- seq(min(a$dates),max(a$dates),len=nDate)
        mov <- DCRW(dd,timeunit="hours")
    }
    Animal(observation = Observation(lon = a$X[2,] + a$Y[2,],
                                     lat = a$X[1,] + a$Y[1,],
                                     dates = a$dates,
                                     locationclass = rep("GPS",
                                                         length(a$dates))),
           measurement = Measurement(model = "n"),
           movement = mov
           )
}

pars <- rbind(c(qlogis(0.9),qlogis(0.9),0,0,0,0),
              c(qlogis(0.6),qlogis(0.6),pi/3,0,0,0))
rownames(pars) <- c("high","low")
varPars <- c(-2,-2)
sdMeas <- log(c(0.1,0.1))

getDist <- function(a,anim1,anim2){
    d0 <- a$dates
    d1 <- anim1$movement$dates
    mu1 <- rbind(approx(d1,anim1$movement$mu[1,],d0)$y,
                 approx(d1,anim1$movement$mu[2,],d0)$y)
    d2 <- anim2$movement$dates
    mu2 <- rbind(approx(d2,anim2$movement$mu[1,],d0)$y,
                 approx(d2,anim2$movement$mu[2,],d0)$y)
    dist1 <- sqrt(colSums((mu1 - a$X)^2))
    dist2 <- sqrt(colSums((mu2 - a$X)^2))
    dist1/dist2
}

getRMSE <- function(a,anim){
    d0 <- a$dates
    d1 <- anim$movement$dates
    mu <- rbind(approx(d1,anim$movement$mu[1,],d0)$y,
                approx(d1,anim$movement$mu[2,],d0)$y)
    RMSE <- sqrt(rowMeans((mu - a$X)^2))
    names(RMSE) <- c("Latitude","Longitude")
    RMSE
}

sdvals <- seq(-6,3,0.5)

for(simnr in 1:200)
    for(scenario in 1:nrow(pars)){
        reslist <- list()
        try({
            for(ii in 1:length(sdvals)){
                simIsOK <- FALSE
                a <-
simModel(250,pars[scenario,],varPars,c(sdvals[ii],sdvals[ii]))
                alb1p0 <- makeAnimal(a,length(a$dates),TRUE)
                fitTrack(alb1p0,fixdrift=TRUE,equaldecay=TRUE,
                         fixmovecor=TRUE,equalvar=TRUE)
                jon1p0 <- makeAnimal(a,length(a$dates),FALSE)
                fitTrack(jon1p0,fixmovecor=TRUE,equalvar=TRUE)
                reslist[[ii]] <- getDist(a,alb1p0,jon1p0)
                rm("alb1p0","jon1p0","a")
```

```
            }
            res <- do.call("rbind",reslist)
            rownames(res) <- sdvals
            save(res,file=paste0("res_dcrw_",rownames(pars)[scenario],
                                 "_evn_",simnr,".RData"))
        })
        rm(list=ls()[-which(ls() %in% c("scenario","simnr","simDt","simModel",
                                        "pars","varPars","sdMeas","makeAnimal",

"getRMSE","getDist","sdvals","reslist"))])
    }
```

S8: R code for the case study

```
library(argosTrack)
library(TMB)
library(covafillr)

Sys.setenv(TZ='UTC')

compile("movement.cpp", CXXFLAGS=paste("-O3",
                                       covafillr::cxxFlags(),
                                       paste0("-I", system.file("include",
package = "argosTrack")))
        )
dyn.load(dynlib("movement"))

## Data
data(subadult_ringed_seal)
d0 <- subadult_ringed_seal
d <- d0[!(d0$lc %in% c("Z")),]

## argosTrack objects
makeObj <- function(nn=Inf,nsi=1){
    obs <- Observation(lon=d$lon,
                       lat=d$lat,
                       dates=as.POSIXct(d$date),
                       locationclass=d$lc)
    ns <- rep(nsi,length.out=2)
    meas <- Measurement(model="t")
    date1 <-
unique(sort(c(as.POSIXct(d$date),seq(min(as.POSIXct(d$date)),max(as.POSIXct(d$da
te)),nn*60*60))))
    mov <- GDCRW(dates=date1)
    anim <- Animal(movement = mov,
                   measurement = meas,
                   observation = obs)
    ## Prepare for TMB
    dat <- anim$getTMBdata()
    date0 <- strptime(strftime(mov$dates,"%y-%m-%d 00:00:00"),"%y-%m-%d %H:%M:
%S")
    dat$knots_mu <- as.matrix(expand.grid(seq(min(d$lat),max(d$lat),len=ns[1]),
                                          seq(min(d$lon),max(d$lon),len=ns[2])))
    dat$rep_lat <- seq(min(d$lat),max(d$lat),0.1)
    dat$rep_lon <- seq(min(d$lon),max(d$lon),0.1)
    dat$p <- 3
    dat$h <- covafillr::suggestBandwith(dat$knots_mu,dat$p)
    dat$d <- 2
    pars <- anim$getTMBparameters()
    pars$mu[1,] <- mean(d$lat)
    pars$mu[2,] <- mean(d$lon)
    pars$knotpar_mu_lat <- rnorm(nrow(dat$knots_mu),0,0.001)
    pars$knotpar_mu_lon <- rnorm(nrow(dat$knots_mu),0,0.001)
    pars$logLambdaLat <- -3
    pars$logLambdaLon <- -3
    map <-
anim$getTMBmap(fixcorrection=FALSE,fixdrift=TRUE,equaldecay=FALSE,fixrotation=FA
LSE,fixmovecor=FALSE)
    obj <- MakeADFun(dat,pars,map,
                     random=c("mu","knotpar_mu_lat","knotpar_mu_lon"),
                     inner.control = list(maxit = 100),
                     DLL = "movement")
    obj
}

obj <- makeObj(1,c(15,15))

obj$fn()
```

```
rp0 <- obj$report(par=obj$env$last.par.best)

opt <- nlminb(obj$par,obj$fn,obj$gr,control=list(iter.max=1000,eval.max=1000))

rp <- obj$report(par=obj$env$last.par.best)



#######################
#### REPORT RESULT ####
#######################

filled.contour(obj$env$data$rep_lon,obj$env$data$rep_lat,t(rp$muLonOut),color.pa
lette=colorRampPalette(c("blue","white","red")),nlevels=100,main="Lon local mean
velocity",
               plot.axes = {
                   lines(rp$slon,rp$slat)
                   points(obj$env$data$knots_mu[,2:1],col=rgb(1,0,0,0.5),pch=16)
               })

filled.contour(obj$env$data$rep_lon,obj$env$data$rep_lat,t(rp$muLatOut),color.pa
lette=colorRampPalette(c("blue","white","red")),nlevels=100,main="Lat mean
velocity",
               plot.axes = {
                   lines(rp$slon,rp$slat)
                   points(obj$env$data$knots_mu[,2:1],col=rgb(1,0,0,0.5),pch=16)
})

#############################
#### GET STANDARD ERRORS ####
#############################


hess <- optimHess(opt$par,obj$fn,obj$gr)

sr <- sdreport(obj,par.fixed=opt$par,hessian.fixed=hess)
sdr <- summary(sr)

pl <- obj$env$parList(par=obj$env$last.par.best)


##############
#### Save ####
##############

save(obj,opt,rp,sr,sdr,hess,pl,file="res_case_study.RData")
```

S9: C++ code for the case study

```
// Save this file as movement.cpp
// Copied and modified from argosTrack: http://github.com/calbertsen/argosTrack

#include <TMB.hpp>
#include <argosTrack.hpp>
#include <covafill/TMB>



using namespace density;

using namespace argosTrack;

template<class Type>
Type objective_function<Type>::operator() ()
{
  //////////
  // DATA //
  //////////

  // Observation related
  DATA_VECTOR(lon);
  DATA_VECTOR(lat);
  DATA_VECTOR(dayOfYear);
  DATA_VECTOR(include);
  DATA_FACTOR(qual);
  DATA_IVECTOR(varModelCode);

  // Movement related
  DATA_VECTOR(dtStates);
  DATA_INTEGER(moveModelCode);
  DATA_INTEGER(nauticalStates);

  // Measurement related
  DATA_SCALAR(minDf);
  DATA_INTEGER(errorModelCode);
  DATA_INTEGER(nauticalObs);
  DATA_VECTOR(splineKnots);

  // Related to more than one
  DATA_IVECTOR(prevState);
  DATA_VECTOR(stateFrac);


  // Residual related
  DATA_VECTOR_INDICATOR(klon,lon);
  DATA_VECTOR_INDICATOR(klat,lat);


  ////////////////
  // PARAMETERS //
  ////////////////

  // Movement related
  PARAMETER_VECTOR(movePars); //Length 2 (first lat then lon) x number of states
  PARAMETER_VECTOR(logSdState);

  // States
  PARAMETER_MATRIX(mu);
  PARAMETER_MATRIX(vel);


  // Measurement related
  PARAMETER_VECTOR(logSdObs);
```

```
PARAMETER_VECTOR(logSdObsExtra);
PARAMETER_MATRIX(logCorrection);
PARAMETER(splineXlogSd);
PARAMETER_VECTOR(knotPars);
PARAMETER_VECTOR(df);  //Length as number of quality classes


// SPline stuff
DATA_MATRIX(knots_mu);

DATA_VECTOR(rep_lat);
DATA_VECTOR(rep_lon);

PARAMETER_VECTOR(knotpar_mu_lat);
PARAMETER_VECTOR(knotpar_mu_lon);

PARAMETER(logLambdaLat);
PARAMETER(logLambdaLon);

DATA_INTEGER(p);
DATA_VECTOR(h);
DATA_SCALAR(d);
covafill<Type> cfLat(knots_mu,knotpar_mu_lat,h,p);
covafill<Type> cfLon(knots_mu,knotpar_mu_lon,h,p);
covatree<Type> ctLat(d, &cfLat);
covatree<Type> ctLon(d, &cfLon);

vector<Type> muUse(2);
muUse.setZero();


// Get state coordinates in latitude/longitude
vector<Type> slon(mu.cols());
vector<Type> slat(mu.cols());

for(int i = 0; i < mu.cols(); ++i){
  slon(i) = mu(1,i);
  slat(i) = mu(0,i);
}

// Transform parameters
vector<Type> varState = exp(Type(2.0)*logSdState);
matrix<Type> varObs(logCorrection.rows(),logCorrection.cols()+1);
matrix<Type> correction = logCorrection.array().exp().matrix();
for(int i = 0; i < varObs.rows(); ++i){
  varObs(i,0) = exp(2.0*(logSdObs(i)));
  for(int j = 1; j < varObs.cols(); ++j){
    varObs(i,j) = exp(2.0*(logSdObs(i)+logCorrection(i,j-1)));
  }
}
matrix<Type> sdObs = varObs.array().sqrt().matrix();

// Variable for negative log-likelihood
Type nll = 0.0;

for(int i = 0; i < knotpar_mu_lat.size(); ++i)
  nll -= dnorm(knotpar_mu_lat(i),Type(0.0),exp(logLambdaLat),true);
for(int i = 0; i < knotpar_mu_lon.size(); ++i)
  nll -= dnorm(knotpar_mu_lon(i),Type(0.0),exp(logLambdaLon),true);

////////////////////////////////
// Create covariance matrices //
////////////////////////////////
```

```
  //Set up covariance matrix for observations
  // Observational distributions
  vector<densities::MVT_tt<Type> > nll_dist_obs(varObs.cols());
  matrix<Type> covObs(2,2);
  covObs.setZero();
  covObs(0,0) = 1.0;
  covObs(1,1) = 1.0;
  vector<Type> obs(2);

  // For argos data
  for(int i = 0; i < nll_dist_obs.size(); ++i){
    covObs.setZero();
    covObs(0,0) = varObs(0,i);
    covObs(1,1) = varObs(1,i);
    covObs(1,0) = 0.0;
    covObs(0,1) = covObs(1,0);
    //ModelCode: 0: t; 1: norm; 2: symmetric hyperbolic
    nll_dist_obs(i) = densities::MVT_tt<Type>(covObs,exp(df(i))
+minDf,errorModelCode);
  }



  ///////////////////////////////////
  // Contribution from first state //
  ///////////////////////////////////


  ///////////////////////////////
  // Contributions from states //
  ///////////////////////////////

  using namespace movement;

  for(int i = 1; i < mu.cols(); ++i){
    // case 6:              // Irregularized Discrete time correlated random
walk on lat+lon
    muUse(0) = ctLat((vector<Type>)mu.col(i-1))(0);
    muUse(1) = ctLon((vector<Type>)mu.col(i-1))(0);
    if(i == 1){
      nll += nll_idtcrw1((vector<Type>)mu.col(i),
                     (vector<Type>)mu.col(i-1),
                     dtStates(i),
                     (vector<Type>)(Type(1.0)/(Type(1.0)+exp(-
movePars.segment(0,2)))),
                     movePars(2),
                     Type(2.0)/(Type(1.0)+exp(-movePars(3))) - Type(1.0),
                     muUse,
                     varState);
      }else{
      nll += nll_idtcrw((vector<Type>)mu.col(i),
                   (vector<Type>)mu.col(i-1),
                   (vector<Type>)mu.col(i-2),
                    dtStates(i),
                    dtStates(i-1),
                    (vector<Type>)(Type(1.0)/(Type(1.0)+exp(-
movePars.segment(0,2)))),
                    movePars(2),
                    Type(2.0)/(Type(1.0)+exp(-movePars(3))) - Type(1.0),
                    muUse,
                    varState);
      }
  }
```

```
  //////////////////////////////////////
  // Contributions from observations //
  //////////////////////////////////////

  for(int i = 0; i < lon.size(); ++i){
    obs.setZero();
    obs(0) = lat(i);
    obs(1) = lon(i);
    if(prevState(i)+1 >= slat.size()){
      obs(0) -= slat(prevState(i));
      obs(1) -= slon(prevState(i));
    }else{
      obs(0) -= stateFrac(i) * slat(prevState(i)) +
      (Type(1.0) - stateFrac(i)) * slat(prevState(i)+1);
      obs(1) -= stateFrac(i) * slon(prevState(i)) +
      (Type(1.0) - stateFrac(i)) * slon(prevState(i)+1);
    }
      nll += nll_dist_obs(qual(i))(obs)*include(i)*klon(i)*klat(i);
  }

  matrix<Type> muLonOut(rep_lat.size(),rep_lon.size());
  for(int i = 0; i < rep_lat.size(); ++i)
    for(int j = 0; j < rep_lon.size(); ++j){
      vector<Type> tmp(2);
      tmp(0) = rep_lat(i); tmp(1) = rep_lon(j);
      muLonOut(i,j) = cfLon(tmp)(0);
    }
  REPORT(muLonOut);
  //ADREPORT(muLonOut);
  // }

  //if(isDouble<Type>::value){
  matrix<Type> muLatOut(rep_lat.size(),rep_lon.size());
  for(int i = 0; i < rep_lat.size(); ++i)
    for(int j = 0; j < rep_lon.size(); ++j){
      vector<Type> tmp(2);
      tmp(0) = rep_lat(i); tmp(1) = rep_lon(j);
      muLatOut(i,j) = cfLat(tmp)(0);
    }
  REPORT(muLatOut);


  ////////////
  // REPORT //
  ////////////

  REPORT(slat);
  REPORT(slon);

  //////////////
  // ADREPORT //
  //////////////

  vector<Type> dfs = exp(df)+minDf;
  ADREPORT(correction);
  ADREPORT(sdObs);
  ADREPORT(dfs);

  return nll;

}
```
